# Identifying Centres of Plant Biodiversity in South Australia

**DOI:** 10.1371/journal.pone.0144779

**Published:** 2016-01-06

**Authors:** Greg R. Guerin, Ed Biffin, Zdravko Baruch, Andrew J. Lowe

**Affiliations:** 1 Terrestrial Ecosystem Research Network, Adelaide-node, School of Biological Sciences, University of Adelaide, North Terrace, Adelaide, South Australia 5005, Australia; 2 Science, Monitoring and Knowledge, Department of Environment, Water and Natural Resources, Adelaide, South Australia, Australia; Chinese Academy of Forestry, CHINA

## Abstract

We aimed to identify regional centres of plant biodiversity in South Australia, a sub-continental land area of 983,482 km^2^, by mapping a suite of metrics. Broad-brush conservation issues associated with the centres were mapped, specifically climate sensitivity, exposure to habitat fragmentation, introduced species and altered fire regimes. We compiled 727,417 plant species records from plot-based field surveys and herbarium records and mapped the following: *species richness* (all species; South Australian endemics; conservation-dependent species; introduced species); *georeferenced weighted endemism*, *phylogenetic diversity*, *georeferenced phylogenetic endemism*; and measures of *beta diversity* at local and state-wide scales. Associated conservation issues mapped were: *climate sensitivity* measured via ordination and non-linear modelling; *habitat fragmentation* represented by the proportion of remnant vegetation within a moving window; *fire prone landscapes* assessed using fire history records; *invasive species* assessed through diversity metrics, species distribution and literature. Compared to plots, herbarium data had higher spatial and taxonomic coverage but records were more biased towards major transport corridors. *Beta diversity* was influenced by sampling intensity and scale of comparison. We identified six centres of high plant biodiversity for South Australia: Western Kangaroo Island; Southern Mount Lofty Ranges; Anangu Pitjantjatjara Yankunytjatjara lands; Southern Flinders Ranges; Southern Eyre Peninsula; Lower South East. Species composition in the arid-mediterranean ecotone was the most climate sensitive. Fragmentation mapping highlighted the dichotomy between extensive land-use and high remnancy in the north and intensive land-use and low remnancy in the south. Invasive species were most species rich in agricultural areas close to population centres. Fire mapping revealed large variation in frequency across the state. Biodiversity scores were not always congruent between metrics or datasets, notably for categorical endemism to South Australia versus *georeferenced weighted endemism*, justifying diverse approaches and cautious interpretation. The study could be extended to high resolution assessments of biodiversity centres and cost:benefit analysis for interventions.

## Introduction

Biodiversity conservation requires application of limited resources, and spatial management priorities are generally based on values, threats and benefits at the level of ecological communities rather than individual species [1]. Identifying areas with high biodiversity value is therefore a fundamental layer of conservation planning, upon which landscape management and its relative costs and benefits can be super-imposed [2,3]. Biodiverse areas are defined on the basis of biological richness or uniqueness, which could relate to the presence of threatened, range-restricted or complementary species and ecological communities [2].

In the Mediterranean Biome, biodiverse landscapes face complex stressors [4] and basic conservation planning requires information on biodiversity values but also, for example, sensitivity and exposure to changes in climatic, land-use, invasion and fire regimes and the cost for return of management actions [1,5]. In South Australia, vegetation remnancy in some regions is low [6] and the climate is predicted to become significantly warmer and drier this century [in the order of +2°C and -10% MAP relative to a 1986–2005 baseline for intermediate emissions pathways; [7]). Although combining information on spatial biodiversity, climate sensitivity and habitat fragmentation can inform conservation priorities [8], it is rarely attempted at macro-ecological scales.

Here, we map coarse resolution plant biodiversity within the context of the state of South Australia using objective, landscape-scale metrics and identify areas of exceptional biodiversity and their associated conservation issues for context. Future extensions to this analysis could include higher resolution analysis over selected areas or in-depth analysis of ecosystem condition, threats and management costs.

### Geopolitical context

**Fig 1:**
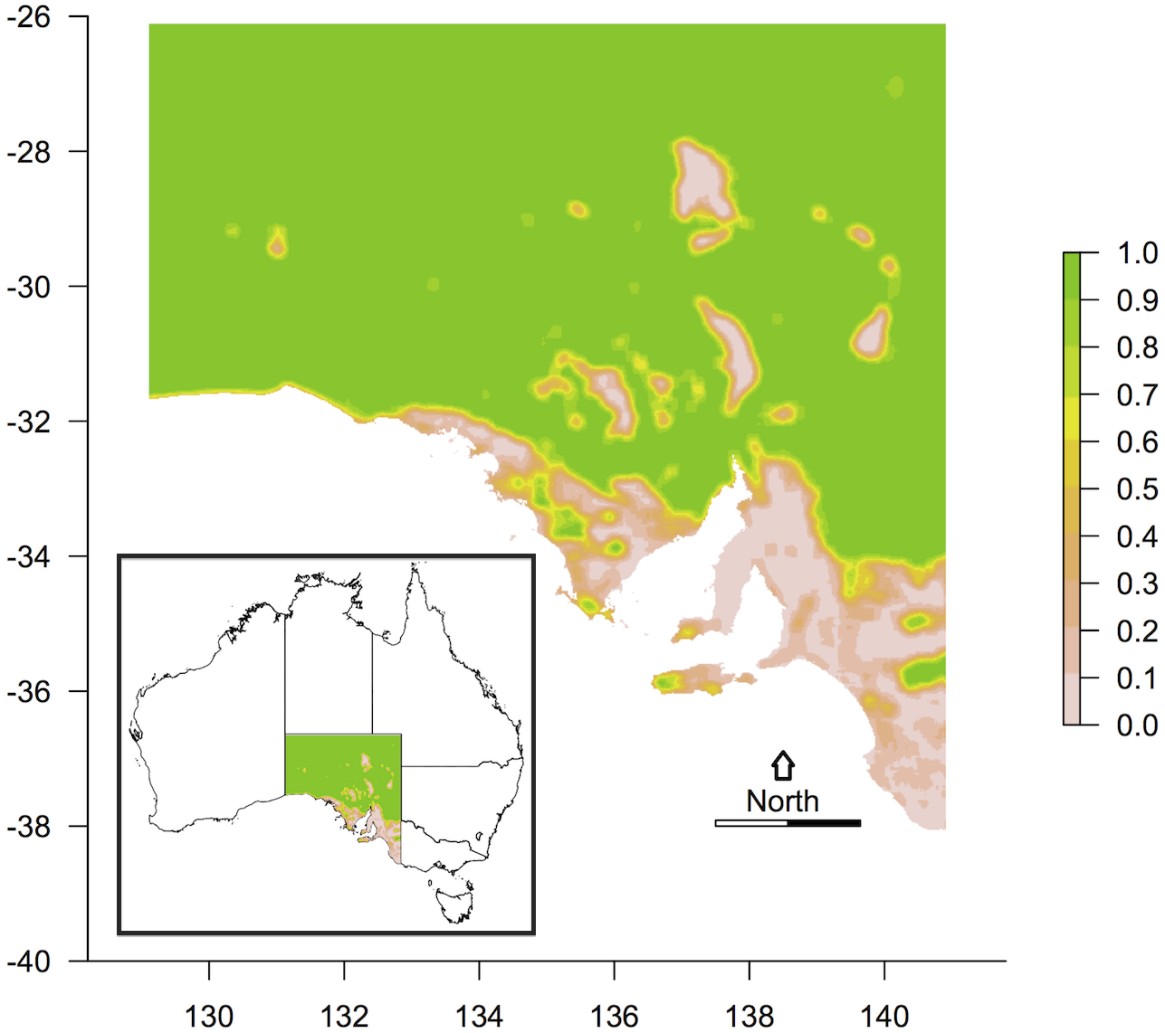
Study region. South Australia on the mainland of the Australian continent (plus its off-shore islands), showing the proportion of remnant native vegetation within a moving window of 25 x 25 km (large areas with no vegetation in the north are salt lakes). Scale bar: 200 km. Inset: context within Australia.

This study is sub-continental and bound within South Australia, occupying the central, southern Australian mainland and off-shore islands (Fig 1). From an ecological perspective, this area is arbitrary, as a number of the state’s bioregions (defined by ‘IBRA’, the Interim Biogeographic Regionalisation for Australia, [9]; Fig A in S1 Appendix) overlap state borders. However, there are two reasons for political bounds: **1**. The existence of a large, yet to date largely untapped, vegetation inventory dataset, the *Biological Survey of South Australia*, which was deliberately implemented to sample the state’s ecological diversity and includes systematically sampled presence/absence data [10]. **2**. Ecosystems are primarily managed at state level in Australia and there is a practical need to provide information for biodiversity conservation in this context.

### Biodiversity assessments for South Australia

Several studies have mapped biodiversity metrics for specific taxa across the Australian continent (e.g. Anura (frogs) [11]; *Acacia* [12]; bryophytes [13]; *Acacia* [14]; *Glycine* [15]; *Eucalyptus* [16], generally revealing idiosyncratic patterns among taxa for South Australia. Crisp *et al*. [17] and Laffan & Crisp [18] used 118,643 Australia-wide herbarium records of 8,560 vascular plants species to identify centres of endemism at a scale of 1° grid cells. For South Australia, they identified the Adelaide–Kangaroo Island area as having high endemism at continental scale. An assessment of the evolutionary uniqueness of the Australian arid biota revealed several South Australian areas as being continental refugia, including the Flinders Ranges and Kangaroo Island [19]. Traill *et al*., [20] analysed data from relevés in the Alinytjara Wilurara Natural Resource Management (AW NRM) region, a desert area in the north-west of South Australia. By partitioning species diversity among *a priori* “major vegetation groups” based on representative plots, they concluded that climate averaged within vegetation groups was not a good predictor. State and Federal Governments have also assessed 'hotspots' for threatened species (see http://www.environment.gov.au/biodiversity/conservation/hotspots/national-biodiversity-hotspots, accessed 14 September 2015) and the regional trajectory of species (see http://www.environment.sa.gov.au/managing-natural-resources/plants-and-animals/Threatened_species_ecological_communities/Regional_significant_projects/Regional_Species_Conservation_Assessment_Project, accessed 14 September 2015).

### Biodiversity sampling issues

Biodiversity mapping is an empirical exercise in representing species inventory data. However, given that large-scale biodiversity mapping studies typically use data from existing sources such as herbarium or museum records collected non-systematically and for a different purpose, biases in the underlying data may influence the results [13,21]. Primary sampling issues for biodiversity mapping include: **1.** uneven and under- sampling, resulting in biased richness estimates; and **2.** that many metrics are correlated with *species richness*. These factors can make it difficult to disentangle metrics from sampling intensity and *species richness*. The absence of information for un-sampled areas is also a major issue for poorly sampled taxa and regions [13]. However, this is less of an issue for this study because, between herbarium and Biological Survey data, the vascular flora of South Australia is relatively well sampled, a significant legacy put to use here.

Plot-based data recording species according to systematic effort criteria make it possible to use the accumulation of species to estimate actual *species richness* from that observed, for example via rarefaction or non-parametric estimators [3,22–24]. However, for the purposes of identifying relative patterns of biodiversity, it is not always necessary to correct metrics for sampling: it may be more relevant to ask whether metrics are higher than expected, given the context of sampling.

### Aims

We aimed to identify centres of high vascular plant biodiversity in South Australia by compiling existing datasets and mapping a suite of quantitative biodiversity metrics (Table 1), making use of significant yet under-utilised data resources. Having identified centres of biodiversity, we aimed to overlay spatial information on associated threats at a similar scale for context. The conservation issues considered were sensitivity to climate change as predicted from spatial analogues, exposure to habitat fragmentation, introduced species and fire regimes.

**Table 1:** Biodiversity metrics mapped for South Australia and associated datasets and tests.

Metric (full)	Metric (short)	Herbarium	Plots	Combined data	Correction or test
*Species richness* of:					
• All species		x	x		Non-parametric estimator
• Native species		x	x		-
• Introduced species			x		-
• Categorical endemics		x	x		Compare observed ~ expected; non-parametric outlier statistics
• Conservation-dependent species		x	x		-
*Georeferenced weighted endemism*	GWE		x		Compare observed ~ expected; non-parametric outlier statistics
*Georeferenced phylogenetic endemism*	GPE		x		Compare observed ~ expected; non-parametric outlier statistics
*Phylogenetic diversity*	PD		x		Compare observed ~ expected; non-parametric outlier statistics
Sørensen dissimilarity, mean of all pairwise comparisons				x	-
Sørensen dissimilarity, mean of comparisons among 5x5 cell moving window				x	-
Correspondence Analysis	CA			x	-
Canonical Correspondence Analysis	CCA			x	Mean difference along constrained axis within 3x3 moving window
Canonical Correspondence Analysis	CCA			x	Slope of local polynomial regression to estimate high turnover and sensitivity
Hierarchical classification analysis	HCA			x	Replacement component of Sørensen dissimilarity only; selected metrics re-calculated for 40 vegetation groups

## Materials and Methods

### Study area

#### Geophysical context

South Australia makes up a sub-continental land area of 983,482 km^2^, comprising approximately 13% of the Australian continent, and consisting predominantly of extensive arid (mean annual rainfall approximately 100–300 mm) plains in the north and low-relief mediterranean-climate (mean annual rainfall approximately 300–1000 mm) regions in the south (source: Atlas of South Australia (http://www.atlas.sa.gov.au), accessed 12 November 2014; Bureau of Meteorology (http://www.bom.gov.au), accessed 12 November 2014). Major landscape features include large salt lakes (the largest, Lakes Gairdner, Eyre, Frome and Torrens), and isolated mountain ranges to 1400 m ASL in the north-west (Central Ranges IBRA bioregion) and to 1100 m ASL along the Mount Lofty–Flinders Ranges (Flinders-Lofty Block and Kanmantoo IBRA bioregions). The state’s coast is characterised by three peninsulas (from west to east: Eyre, Yorke, Fleurieu) and off-shore islands, including Kangaroo Island, Australia’s third largest, at 4,405 km^2^ (Fig A in S1 Appendix).

#### Vegetation condition

Eighty-seven percent of the state’s land area is arid and retains 96% of its native vegetation, although there is extensive use as rangelands [6,25]. The remaining 13% of land area in the mediterranean-climate south retains 4–26% of native vegetation and, although broad-acre clearing has been prohibited since 1991, illegal and legal clearing of degraded vegetation continues [6].

Despite progressive increases to the area of land under formal conservation protection, vegetation condition in many areas is in decline due to altered fire regimes, introduced pests and poor recruitment [6,26]. Management of land-use in remnants is also an on-going issue. Of native vegetation sites in the Northern and Yorke Natural Resource Management region, for example, 60% have poor control of livestock grazing [6]. In the Adelaide–Mount Lofty Ranges region there has been peri-urban land-use intensification, such as the conversion of lightly grazed paddocks to vineyards [25].

### Datasets

#### Species data

A set of 330,004 individual records of 3,083 vascular plant species from 14,328 systematically surveyed field plots across the state was obtained from the *Biological Survey of South Australia*, or 'BSSA' [27,28]. BSSA plots were established to systematically sample and document variation in ecological communities in the context of their environments to provide information for reserve planning and the identification of areas with high biological significance [10], although such an analysis has never been conducted at state level. The standard plot size was 30 x 30 m, although some plots were larger in arid areas, and some earlier plots were 10 x 10 m.

BSSA data were supplemented with plot-based data from the Terrestrial Ecosystem Research Network’s (TERN) ‘AusPlots Rangelands’ [29]. Data were subsetted to plant species vouchered following exhaustive visual searches of 27 one hectare plots in the arid zone of South Australia, which is generally under-sampled, resulting in an additional 967 records of 231 species [30].

In addition to these datasets, herbarium records were obtained through Australia’s Virtual Herbarium with the following filters: state of South Australia as the spatial extent; records from the State Herbarium of South Australia; spatially valid records; no uncertain specimen determinations; identified to species or lower taxonomic level [31]. Records for genera previously belonging to family Chenopodiaceae (now Amaranthaceae) were manually extracted, as the above filters excluded them due to missing classification fields. The resulting dataset consisted of 396,446 records of 4,501 species.

Fields associated with species names in the South Australia census of vascular plants [32; electronic version updated June 2014] were matched with species records to subset data to native or introduced species and species considered endemic to South Australia (updated 2014, P.J.Lang pers. comm.).

#### Environmental and fire history data

The following map layers were obtained via ‘MapLand’ (Department of Environment, Water and Natural Resources, SA): polygons of extant native vegetation across South Australia (for calculation of habitat fragmentation indices); fire history layers consisting of polygons of fire events. Raster maps of bioclimatic variables were obtained using tiles from WorldClim [33] trimmed to the extent of South Australia.

### Mapping resolution

Biodiversity was mapped over 0.25° grid cells, which was found to be a reasonable balance between high enough resolution for interpretation and a low enough resolution to capture a representative sample of species records. Biodiversity rasters were subsequently smoothed at a resolution of approximately 1 km^2^ grid cells (representing values for the surrounding area of 0.25°). Smoothing at a finer resolution allowed areas outside of the coastline to be trimmed and provides a more realistic output that minimises artificial differences between adjacent cells, although mainly visual. We recognise that at this scale there is likely to be variation within grid cells in levels of biodiversity and vegetation remnancy, for example.

### Sampling corrections

Sampling issues included uneven intensity, biases in locations and taxa and correlation between *species richness* and other metrics. In some instances, we calculated raw metrics, with only qualitative interpretation of sampling influence. This was the case for herbarium data which, despite in creased taxonomic and spatial coverage over plots, are more biased towards highways and populations centres, have fewer records representing occurrences of common species and are more problematic to correct for sampling intensity [34–36]. For total *species richness* estimated from plot data, we employed the bootstrap non-parametric richness estimator to boost estimates of richness in under-sampled grid cells [37,38]. Non-parametric estimators were chosen among alternatives such as rarefaction as they are computationally efficient, although we recognise these estimates are likely still biased in cells with very low sampling [23].

For metrics associated with endemism or phylogenetic diversity, we used non-parametric tests, described below along with the specific metrics, as to whether observed values were different to that expected, given the number of species recorded, a method that accounts for the influence of both under-sampling and *species richness* itself [27].

For selected metrics, we calculated a 3 x 3 cell moving window mean to smooth artificial distinctions between adjacent cells due to spatial errors or sampling [13]. The moving window provides estimates for un-sampled grid cells based on the assumption that they will be similar to their immediate neighbours, although we acknowledge this may occasionally produce anomalies.

### Biodiversity metrics

#### Species metrics

*Species richness* was calculated using plot-based data for all species; native species only; introduced weed species only, conservation-dependent species only (defined as those species with a rating under the South Australian National Parks and Wildlife Act 1972) and known South Australian endemics only (*endemic richness*). Herbarium records were used to map occurrences of Buffel Grass (*Cenchrus ciliaris* L.), a weed previously identified as having a disproportionate impact in ecosystem function and native species diversity in arid Australian ecosystems [39,40].

*Endemic richness* was compared to the null model that presences of endemic species are random, given observed *species richness*, and do not represent any special pattern. The aim was to distinguish concentrations of endemic species from areas of higher *species richness* that, by default, may have higher absolute numbers of endemics. Observed *endemic richness* species was compared to that in 1,000 replicate random draws of species from the overall pool (with sampling probabilities weighted by observed species frequencies), and calculating non-parametric two-tailed statistical significance (considered statistically significant if lying within or beyond the upper or lower 2.5%), and a continuous outlier metric (the factor of the interquartile range by which observed values out-lie the median of the null distribution).

*Georeferenced weighted endemism* (GWE: *species richness* weighted by restriction in extent of occurrence; [41]) was mapped using the implementation of Guerin *et al*., [27] with the distance spanning species occurrences as weights. Raw scores were compared to 1,000 replicated random draws (null distribution) to calculate statistical significance and categorical and continuous outlier metrics as for *endemic richness* (method also described in Guerin *et al*., [27]). GWE included native species only, as geographically restricted introduced species are not regarded as having conservation value.

#### Phylogenetic metrics

A phylogenetic tree of all plant species in the dataset was generated from Phylomatic Version 3 (http://phylodiversity.net/phylomatic/) and tree R20120829 [42]. Node age constraints were applied after Wikström *et al*.,[43] along with branch-length adjustment using the bladj algorithm, a simple estimator that spaces nodes evenly between age constraints [44].

*Phylogenetic diversity* (PD; [45]) was calculated by summing the branch-lengths of the phylomatic tree after pruning to species occurring in each grid cell. *Georeferenced phylogenetic endemism* (GPE: phylogenetic diversity weighted by restriction in the extent of occurrence of branches; [41,46] was calculated using the implementation of Guerin & Lowe [47] with the maximum distance spanning the geographical range of branches for weights.

Raw PD and GPE scores were tested as to whether they were higher or lower than expected, given observed *species richness*, with a non-parametric method equivalent to that described for *endemic richness* and outlined for phylogenetic metrics in Guerin & Lowe [47].

### Turnover analysis

#### Datasets

Species records from plot and herbarium data were combined to obtain the most complete possible representation of species composition (as opposed to unbiased estimates of *species richness* or other alpha biodiversity metrics). With these records, we generated a matrix of species presence/absence in grid cells for beta diversity analysis.

#### Beta diversity and climate sensitivity

We calculated pairwise Sørensen dissimilarities for grid cells. As a measure of compositional uniqueness in the context of the whole study region, mean dissimilarities for each grid cell compared to all other cells were mapped, excluding cells with fewer than 20 observations, for which dissimilarities were inflated. As a measure of more local heterogeneity, mean dissimilarities were also calculated among all cells within a 5 x 5 cell moving window.

We applied Correspondence Analysis (CA) to the occurrence matrix and mapped out scores for the first axis. The first CA axis is frequently reported, and visually appeared in this case, to correlate with major latitudinal climatic gradients. We explored this further with scatterplots and calculation of correlation coefficients (using Spearman's rho because the relationships appeared monotonic but obviously non-linear) for axis scores and bioclimatic gradients. The variable ‘mean temperature of the hottest month’ was this most highly correlated with axis scores (ρ = -0.92), followed by 'mean annual rainfall' (ρ = 0.81). We therefore repeated the ordination analysis using Canonical Correspondence Analysis (CCA) constrained by ‘mean temperature of the hottest month’. Subsequently, we calculated the mean distance along the resulting CCA axis among a moving window of 3 x 3 cells to highlight areas where composition is changing more rapidly along this axis, and used local polynomial regression (‘loess’) to explore non-linear relationships between weighted average scores (based on species scores for sites and not constrained to be linear combinations of constraining variables) and this gradient. Regions with the highest slope (change in axis score over change in temperature variable) were interpreted as having a species composition more sensitive to spatial changes in temperature and expected temporal changes [7,48]. CCA has been shown to be a robust method because, unlike CA, it is not prone to artefacts such as the arch effect [48, 49].

### Hierarchical classification

Species composition was used to classify major vegetation groups based on hierarchical clustering with complete linkage agglomeration and Sørensen replacement (not richness-difference) dissimilarities [50]. Grid cells were excluded from training the classification if there were fewer than 20 records within them, to avoid biases and difficulty classifying cells.

The classification was cut into 40 groups, which, although arbitrary, gave a reasonable number of evenly sized groups. Groups were mapped onto a raster layer with a simple smoothing function to account for under-sampled grid cells by assigning the most frequent vegetation group within a 3 x 3 moving window. The five most frequent species in constituent grid cells were identified for each group.

We matched the classification to GPE to represent the biodiversity value of the 40 vegetation groups because GPE is increased by elements of *species richness*, range restriction and phylogenetic diversity. Cells in each group were assigned the mean GPE score. Secondly, to represent vegetation groups or habitats impacted by weeds, we calculated the richness of introduced species recorded within each group.

### Habitat fragmentation and fire layers

We converted remnant native vegetation polygons to a ~1 km^2^ resolution raster, and for each grid cell calculated the proportion of native vegetation within a 25 x 25 km moving window, to represent the fragmentation level of the habitat matrix surrounding each location. The aim was to identify the overall remnancy level at coarse scale to identify heavily cleared and fragmented landscapes, whereas finer resolution mapping would be needed to accurately determine habitat configuration at more local scales. A map of fire frequency was generated and rasterised then used to visually identify the intersection of fire prone ecosystems with high biodiversity. There was no intention to analyse whether fire regimes were appropriate from a biodiversity perspective, the aim was simply to identify whether fire management interacted with any identified biodiversity centre.

### Software

Analyses were conducted with custom scripts in base R [51], and using functions from packages including ‘ape’, ‘vegan’, ‘raster’, ‘labdsv’, ‘maptools’, ‘simba’, ‘plyr’ and the functions of Guerin *et al*., [27] and Guerin & Lowe [47]. The species list for phylomatic was prepared with a custom script that attached family names via automated searches of the Atlas of Living Australia (www.ala.org.au) and The Plant List (www.theplantlist.org).

## Results

### Sampling intensity

A map of the spatial density of herbarium collections revealed high spatial coverage but a significant bias to locations near the capital city of Adelaide (Fig B in S1 Appendix). The spatial coverage of biological survey plots was less complete but more even in intensity.

### Biodiversity metrics

**Fig 2:**
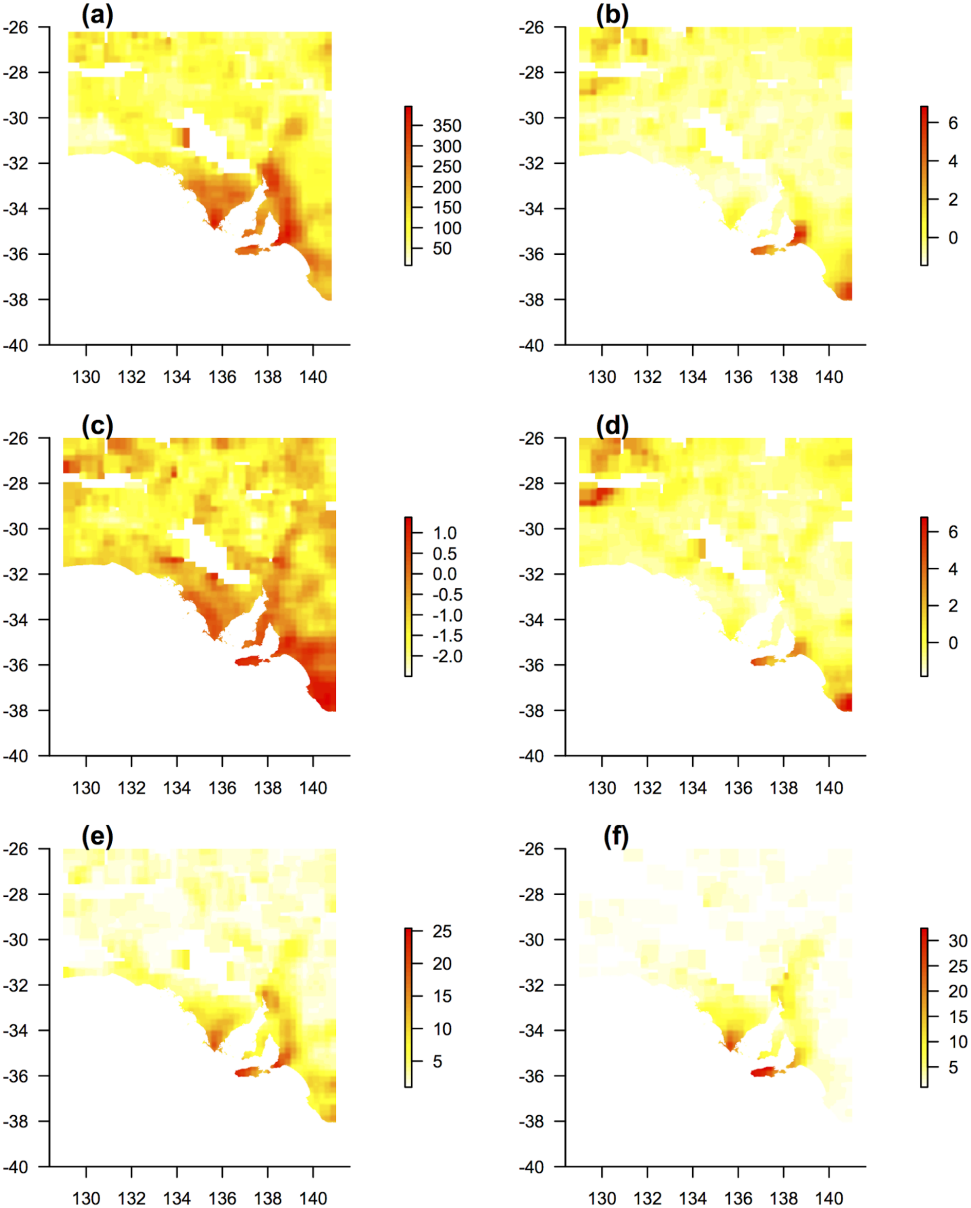
Maps of measured biodiversity metrics. Maps are smoothed at a resolution of 1 km^2^ with values given per surrounding 0.25° x 0.25° area: (a) Estimated *species richness* based on non-parametric estimator (plot data); (b) *georeferenced weighted endemism* of native species (plot data)–continuous outlier metric; (c) *phylogenetic diversity* (plot data)–continuous outlier metric; (d) *georeferenced phylogenetic endemism* (plot data)–continuous outlier; (e) *species richness* of conservation-dependent species (plots); (f) *species richness* of categorical South Australian endemics (plot data).

*Species richness* was generally higher throughout the southern agricultural areas than in the arid north (Fig 2a). The Mount Lofty Ranges, east of Adelaide, had very high *species richness* with herbarium data coinciding with large numbers of collections in this region (Figs B and C in S1 Appendix).

Conservation-dependent *species richness* differed according to dataset. Herbarium data suggested Adelaide-Mount Lofty Ranges and Lower South East were the highest, followed by western Kangaroo Island, southern Flinders Ranges, and lower Eyre Peninsula. Plot data suggested a more even richness among those locations, but lower richness in the Lower South East (Fig 2e and Fig C in S1 Appendix).

Concentrations of (categorical) *endemic richness* were evident on Adelaide-Mount Lofty Ranges, Kanagaroo Island, lower Eyre Peninsula and scattered areas of the Flinders Ranges. The highest absolute numbers, and the most outlying and statistically significant concentrations, were on lower Eyre Peninsula and Kangaroo Island (Fig 2f and Fig D in S1 Appendix.

GWE was higher than expected in several regions: Adelaide–Mount Lofty Ranges, western Kangaroo Island, Lower South East, and two locations in the far north-west of the state: 1. The northern (ranges) section of the APY (Anangu Pitjantjatjara Yankunytjatjara) lands, near the northern state border; 2. The Mamungari Conservation Park, near the western state border (Fig 2b and Fig E in S1 Appendix). GWE was statistically, but barely, higher than expected for the lower Eyre Peninsula. A large area across the middle of the state had slightly lower than expected endemism.

Raw PD had a similar pattern to corrected *species richness* with higher values in southern agricultural regions (Fig F in S1 Appendix). When compared to null expectations, phylogenetic diversity was statistically—but barely—higher than expected in many locations in the south (Fig 2c and Fig F in S1 Appendix). PD was above median expectations in the north-west but this was not statistically significant. A large area of the arid interior had slightly lower than expected phylogenetic diversity (statistically significant).

GPE was concentrated, and higher than expected, in the same locations as for GWE, although with higher relative scores for the north-west (Fig 2d and Fig G in S1 Appendix). These areas were statistically significant, along with a small number of other areas that were only slightly above expected values. Large areas mostly through the middle of the state had slightly lower than expected phylogenetic endemism.

### Turnover analysis

Beta diversity based on state-wide dissimilarity appeared to be biased for under-sampled grid cells, as the poorly sampled areas scored higher than those with better access (Fig H in S1 Appendix). Notwithstanding, the general pattern was scores that were low for the arid zone and increased to the south. Moving-window analysis revealed the opposite pattern, whereby the southern agricultural areas had relatively low beta diversity, whereas there was high beta diversity across the entire arid zone in the north (Fig H in S1 Appendix).

**Fig 3:**
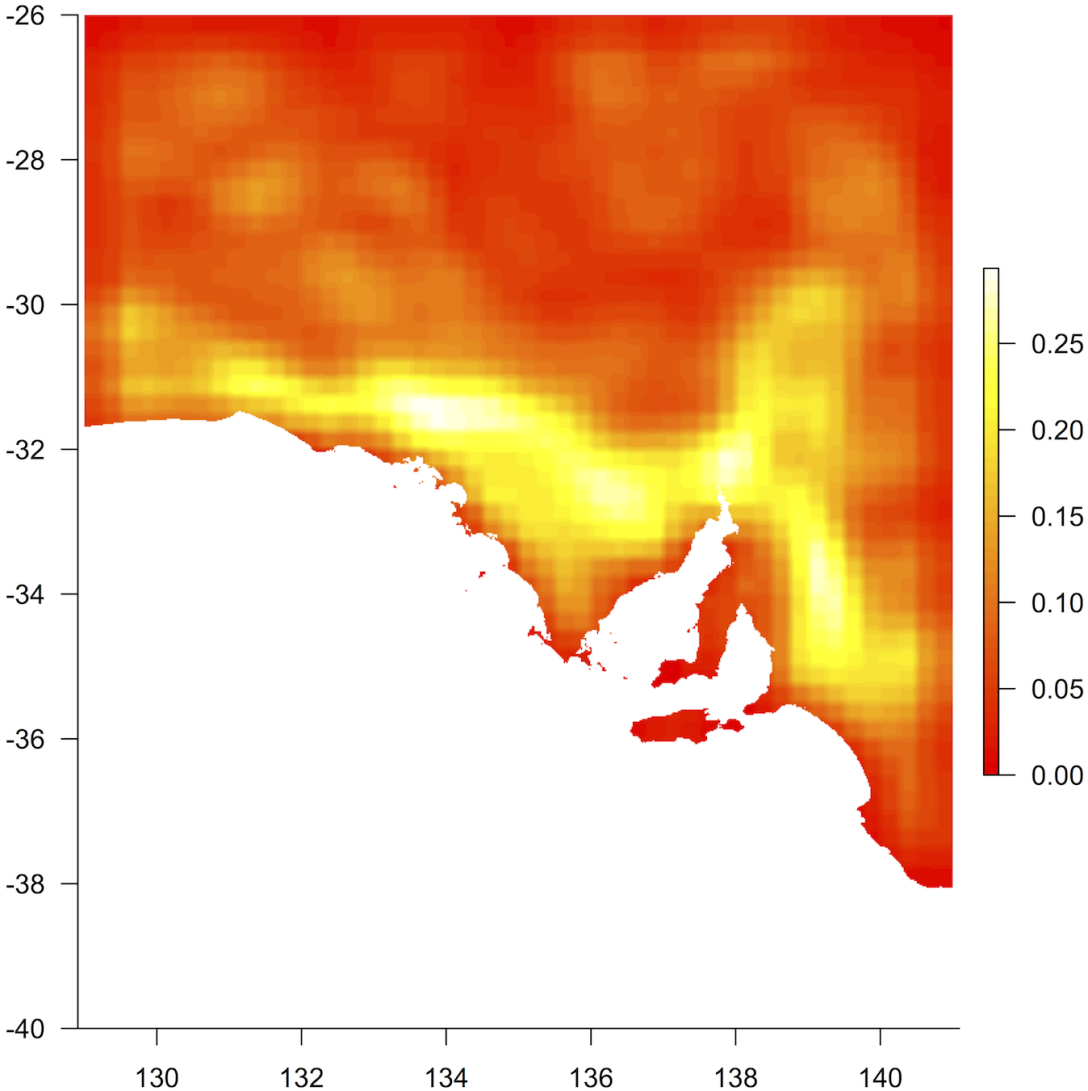
Beta diversity. Here beta diversity is represented by the mean change in a Canonical Correspondence Analysis axis coordinate within a moving window of 3 x 3 grid cells, when this axis is constrained by ‘mean maximum temperature of the hottest month’ (combined data). Higher values represent more rapid turnover along the compositional axis.

**Fig 4:**
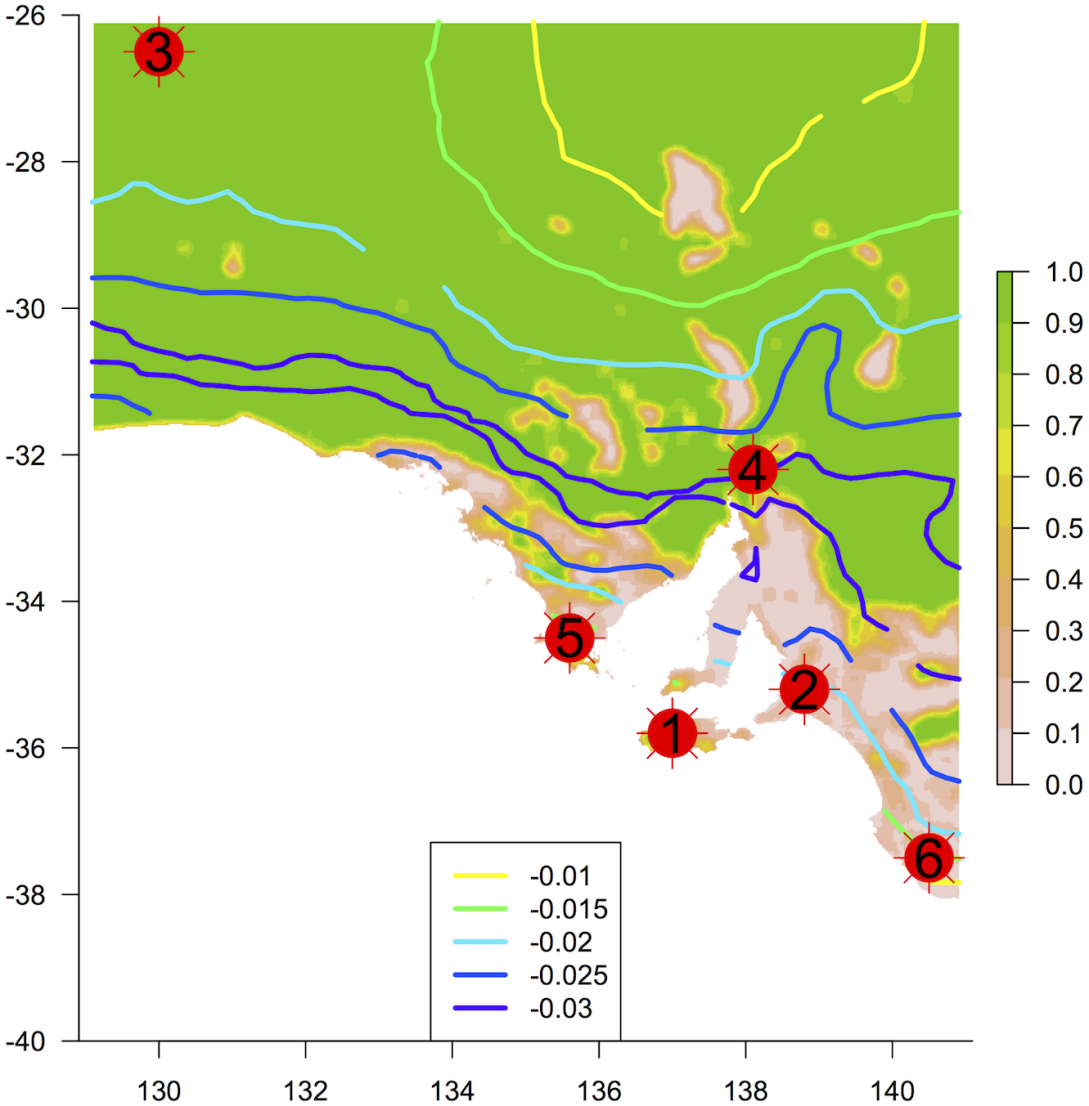
Identified centres of plant biodiversity. The base raster map shows the proportion of remnant vegetation in a moving window (see Fig 1). Contours show the rate of change in species composition with spatial increases in temperature (predicted from a locally weighted nonlinear regression of the first CCA axis), where more negative slopes indicate higher climate sensitivity. Circles show locations of 6 centres referred to in Table 2. See Table 2 for more detail on the identified centres.

Correspondence analysis returned a primary compositional axis running approximately north–south through the state (Fig H in S1 Appendix). When this ordination was repeated constrained by the ‘mean maximum temperature of the hottest month’ climate variable in a CCA analysis, change along the constrained axis within a moving spatial window showed an area overlapping the boundary between arid and mediterranean regions of the state where turnover was higher (Fig 3). The largest rate of change in the compositional axis modelled along the temperature gradient also corresponded to this ecotone (Fig 4).

### Classification analysis

Classification analysis identified 40 specified vegetation clusters and their most frequent species (Fig I and Table A in S1 Appendix). Mean GPE for grid cells assigned to the clusters showed that high scoring clusters were restricted to southern coastal areas of the state. Clusters with the highest GPE were found on Kangaroo Island–Fleurieu Peninsula(–south-east) followed by the lower Yorke Peninsula–Adelaide-Mt Lofty Ranges cluster.

### Exposure to habitat fragmentation, fire and invasive species

Fragmentation mapping clearly showed a dichotomy in land-uses, with the arid north nearly completely vegetated, whereas the southern areas have been reduced to 0–30% vegetation (Fig 1). Highly fragmented landscapes coincided with most, but not all, areas of high biodiversity.

**Fig 5:**
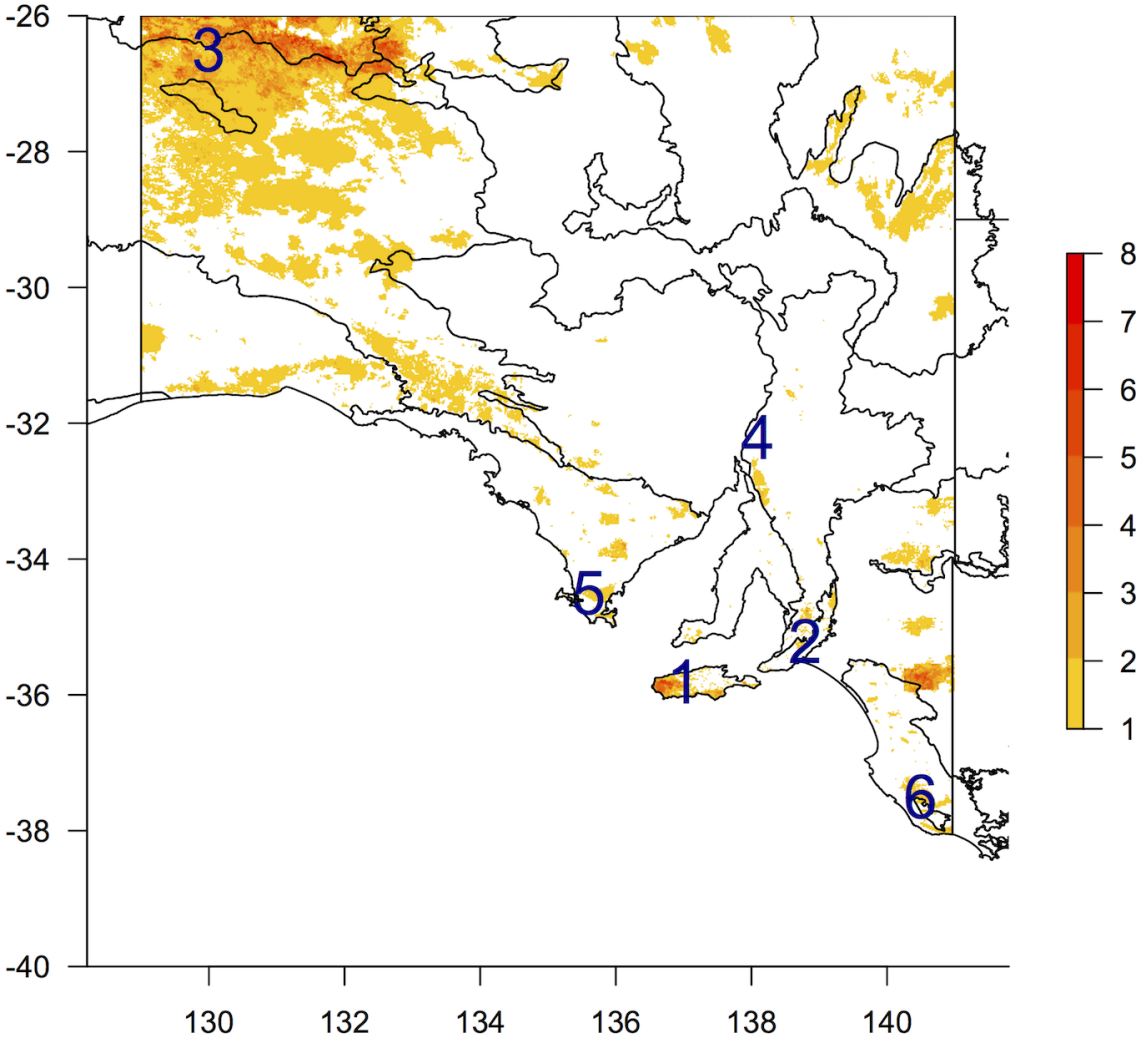
Number of recorded significant fires. Base map shows IBRA bioregions (Fig A in S1 Appendix). Numbers show locations of 6 centres referred to in Table 2.

Fire mapping highlighted areas prone to higher fire frequency (Fig 5). While most patches of remnant native vegetation in higher rainfall areas of the state have been subject to recorded fires, high fire frequency areas of note include the APY lands (NW South Australia) and western Kangaroo Island. Low fire frequency areas generally corresponded to vegetation that is either too sparse or fragmented to carry significant fires.

**Fig 6:**
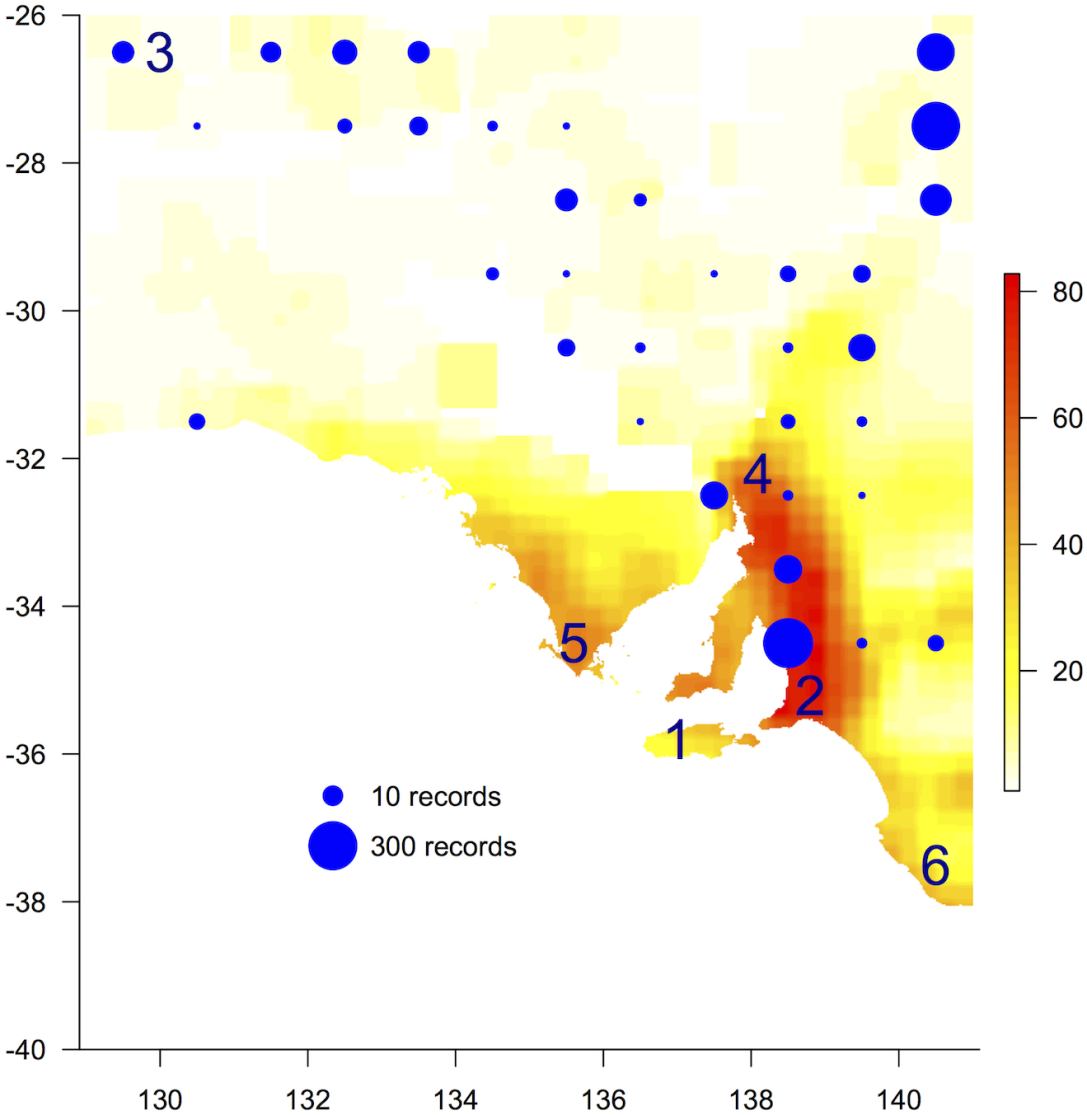
*Species richness* of introduced plant species (plot data). Circles represent the density of individual records of Buffel Grass (*Cenchrus ciliaris* L.) within 1° grid cells on a logarithmic scale. Note, the number of records is influenced by sampling biases and does not directly indicate abundance. Numbers show locations of centres referred to in Table 2.

Richness of introduced species based on plots (which sampled remnant vegetation) was concentrated in agricultural areas close to population centres, most notably high from the Fleurieu Peninsula north to the southern Flinders Ranges and to a lesser extent southern Eyre and York Peninsulas, while Kangaroo Island and the south east of the state were notably lower (Fig 6 and Fig C in S1 Appendix). Weed richness was high (i.e. ~>>200 spp.) for several vegetation clusters covering diverse areas such as the Nullarbor Plain (western coast-line), western Eyre Peninsula and lower Yorke Peninsula, while clusters in the arid zone had few weed species (i.e. ~<<100 spp.), excepting the northern Flinders Ranges and the dunefields of the Great Victoria Desert bioregion, which had medium weed richness (i.e. ~100–150 spp.; Fig I in S1 Appendix). Recorded Buffel Grass occurrences were concentrated in the far north-east and north-west of the state as well as in the vicinity of major transport corridors to the capital city of Adelaide in the south (Fig 6).

### Centres of biodiversity

We identified six locations as centres of biodiversity (in no particular order; Table 2; Fig 4): **1.** Western Kangaroo Island; **2.** Southern Mount Lofty Ranges; **3.** APY Lands (NW South Australia); **4.** Southern Flinders Ranges; **5.** Southern Eyre Peninsula; **6.** Lower South East. Conservation issues associated with these locations were determined via the maps of climate sensitivity, habitat fragmentation, weed diversity and fire frequency (Table 2).

**Table 2:** Identified centres of plant biodiversity in South Australia.

Region	Biodiversity assets	Associated conservation issues
1.Western Kangaroo Island	1. Higher than expected GWE and GPE; 2. Concentrations of conservation-dependent species and categorical endemics; 3. High *species richness*	1. High fire frequency
2. Southern Mount Lofty Ranges	1. Higher than expected GWE and GPE; 2. Concentrations of conservation-dependent species; 3. High *species richness*	1. High levels of habitat fragmentation; 2. Many introduced species
3. APY lands (NW South Australia)	1. Higher than expected GWE and GPE	1. High fire frequency; altered fire regimes; 2. Management of Buffel Grass invasion
4. Southern Flinders Ranges	1. Concentrations of conservation-dependent species; 2. High *species richness*	1. High grazing disturbance levels; 2. High climate sensitivity
5. Southern Eyre Peninsula	1. Concentrations of conservation-dependent species and categorical endemics; 2. High *species richness*	1. High levels of habitat fragmentation; 2. Many introduced species
6. Lower South East	1. Higher than expected GWE and GPE; 2. Concentrations of conservation-dependent species (herbarium data)	1. High levels of habitat fragmentation

## Discussion

We set out to provide basic biodiversity information collectively on some 4,500 species of vascular plants across South Australia. This analysis of species occurrences used intensively sampled biological data and a suite of sophisticated, objective metrics to map areas of high diversity. Six localised areas of the state were clearly highlighted (Fig 4). Although neither conservation prioritisation per se nor detailed assessment of ecosystem condition or trajectory were our primary aim, mapping of major conservation issues showed that each biodiversity centre is associated with issues such as habitat fragmentation and weed invasion at coarse scales.

The southern Mount Lofty Ranges scored highly for unique species and overall high diversity, but has been subjected to the most disturbance since European settlement, with high levels of weed invasion and habitat fragmentation, which suggests a higher resolution assessment is warranted. The western side of Kangaroo Island has perhaps the most significant plant biodiversity in South Australia, scoring consistently highly, but has higher reservation levels and lower incidences of weed species.

This study was made possible by the wealth of information on the distribution of species within the state including intensive herbarium sampling and a Biological Survey program that has systematically recorded vegetation across the state. These data allowed the use of numerical methods highlighting different aspects of biodiversity that can be independently repeated, without reliance upon expert opinion or modelling to fill gaps.

### Comparison of metrics

Crisp *et al*., [17] concluded that the Adelaide–Kangaroo Island region of South Australia had high endemism at a continental scale. In our study, areas highlighted for endemism depended somewhat on the method. GWE highlighted the Lower South East and north-west of South Australia in addition to Kangaroo Island and Adelaide-Mount Lofty Ranges. The former locations are geographically unique within South Australia and lie in the corners of political borders. By comparison, categorical *endemic richness* highlighted Kangaroo Island and lower Eyre Peninsula in particular. Both metrics convey relevant information for conservation in a state context: GWE for highlighting species with restricted ranges in the state (although potentially biased by distributions overlapping state borders); *endemic richness* for highlighting species only found in the state (although a function of political boundaries and potentially biased by species with restricted ranges occurring in the middle of the state being more likely endemic to it).

The APY lands have areas of topographical variation in the Tomkinson, Mann, Musgrave, Everard, and Indulkana Ranges and around granite outcrops, providing refugia such as gorges and high altitude summits [52]. The GWE and GPE results highlight the value of conservation management in the northern APY lands. It is already recognised that fire regimes and the spread of invasive species including feral animals and Buffel Grass need to be managed in partnership with traditional owners to promote ecological outcomes [26,53]. We are cautious about the high GPE scores immediately south of the APY lands within the Mamungari Conservation park (Great Victoria Desert bioregion) because sampling has been limited by access, and in any case, threat levels and therefore need for management are relatively low given the park’s UNESCO World Biosphere Reserve status.

Beta diversity highlighted no particular location, and which general region scored higher depended on whether comparisons were state-wide or local. We interpret the high heterogeneity of species composition along a major ordination axis across the region separating the arid and mediterranean biomes as an ecotone [48].

### Associated conservation issues

#### Climate sensitivity

High spatial turnover in species composition along a major latitudinal climatic gradient, as evident in our data across an ecotone region, can be interpreted as potential high sensitivity to temporal climate change [54]. Space-for-time substitution of course assumes that the spatial sensitivity of species composition to temperature will translate to sensitivity to temporal changes in climate. Mean daily summer maximum temperatures across this region are predicted to rise by two degrees this century over 1986–2005 levels, based on median predictions of 15 models under an intermediate emissions pathway [7]. This is significant because a spatial difference of two degrees is associated with significant shifts along an associated species composition axis.

All but one of the identified centres of biodiversity, the southern Flinders Ranges, occur outside of the area with the highest climate sensitivity, but climate adaptation may be a major concern for ecosystem management in this zone and a consideration for less sensitive areas. It is important to note that there are other axes of compositional variation that may not respond at all to climate change, and that the translation of spatial changes in vegetation along climatic gradients to temporal change is an assumption that needs to be tested through long term monitoring.

#### Habitat fragmentation

The dichotomy between extensive and intensive land-uses between the state’s arid/mediterranean biomes presents contrasting management issues among the identified biodiversity centres. The lower Eyre Peninsula, Adelaide-Mount Lofty Ranges and Lower South East centres have been the most extensively cleared, suggesting addressing the impacts of habitat fragmentation may be an important concern in these areas. In contrast, extensive land management issues such as invasive species and fire regimes are critical for the APY lands, where vegetation condition rather than remnancy is the key issue. The mapping of habitat presence was relatively coarse at a resolution of 1 km^2^. A future extension of this study would be to map biodiversity metrics and landscape characteristics, including habitat remnancy and configuration at much higher resolution over identified biodiversity centres.

#### Fire regimes

The most extensively fire prone region according to frequency mapping is the APY lands. Given low levels of habitat fragmentation, maintaining appropriate fire regimes across this area is a primary concern for conservation. Fire management is also relevant to managing remnant vegetation in the south of the state, where there are localised areas of high fire frequency, notably western Kangaroo Island. Our mapping recorded fire frequency but not intensity or season, nor how appropriate fire regimes have been for biodiversity conservation to date. Future extensions to work would need to assess both the most appropriate fire regimes for maintaining biodiversity but also how fire regimes may be changing. The present study gives a broad-brush indication of the main areas in which fire is an issue.

#### Invasive species

Introduced *species richness* was higher in southern agricultural areas receiving higher rainfall and in major population corridors and this may translate to pressure on the maintenance of native species. A notable exception is Kangaroo Island, which has relatively low weed richness despite a mesic climate, perhaps due to isolation from mainland propagule sources and lower levels of habitat fragmentation.

Weed richness does not account for abundance or directly provide information on whether weed diversity correlates negatively with native diversity. Particular introduced weeds may disproportionately contribute to conservation impacts. Buffel Grass, in particular, has been shown to invade relatively unperturbed habitats and to have measurable impacts on native species diversity and fire regimes [39,40,55,56]. The major impact zone for Buffel Grass at present is the north-west of the state, where feedbacks with fire regimes are an issue.

## Conclusions

We identified six centres of high plant biodiversity across South Australia, based on metrics mapped from plot and herbarium data. We also mapped their spatial interaction with climate sensitivity, habitat fragmentation, fire frequency and weed *species richness*. Areas highlighted were not always congruent among metrics or datasets, which affirms our approach of using a suite of complementary metrics and alternative data sources to increase confidence. Herbarium data captured more species and locations but were more uneven in intensity. Highlighted centres of endemism differed between categorical and weighted metrics, perhaps due to contrasting biases caused by political boundaries.

Species composition in the arid-mediterranean ecotone was most sensitive to climate, although only one biodiversity centre—the southern Flinders Ranges—was located in this zone. Three of the biodiversity centres coincided with highly fragmented vegetation, and four with high weed diversity. For the APY lands, management of fire and Buffel Grass were the main associated issues.

Practical application of these results would benefit from higher resolution assessments within identified biodiversity centres and information on the cost:benefit ratio of intervention, taking into account potential biodiversity/area returns but also costs such as land purchase, lost production opportunity and the market price for management actions.

## Supporting Information

S1 Appendix Additional maps and tables of biodiversity metrics. (PDF)
