# Supplementary material for: Identifying Centres of Plant Biodiversity in South Australia
Source: PLoS One. 2016 Jan 6;11(1):e0144779. doi: 10.1371/journal.pone.0144779 (PMC4703376; doi:10.1371/journal.pone.0144779)
Supplement: S1 Appendix — (PDF) [file pone.0144779.s001.pdf]

Identifying centres of plant biodiversity in South Australia

Greg R. Guerin, Ed Biffin, Zdravko Baruch, Andrew J Lowe.

S1 Appendix. Additional maps and tables of biodiversity metrics.

Figures A–I

Table A

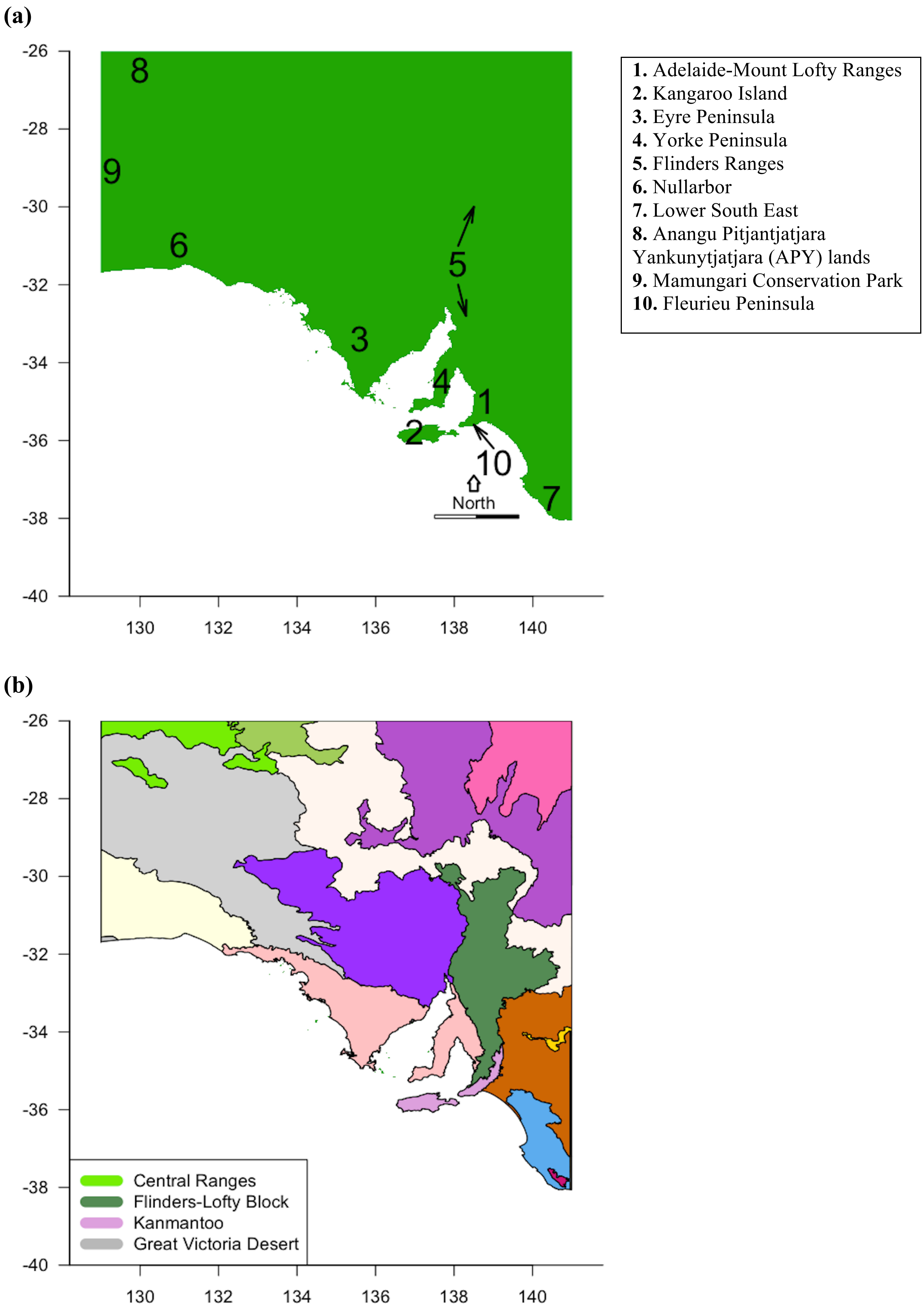

Figure A. Supplementary location maps of South Australia showing: **(a)** the approximate location of place names mentioned in the main text. Scalebar 200 km; **(b)** IBRA bioregions mentioned in the main text.

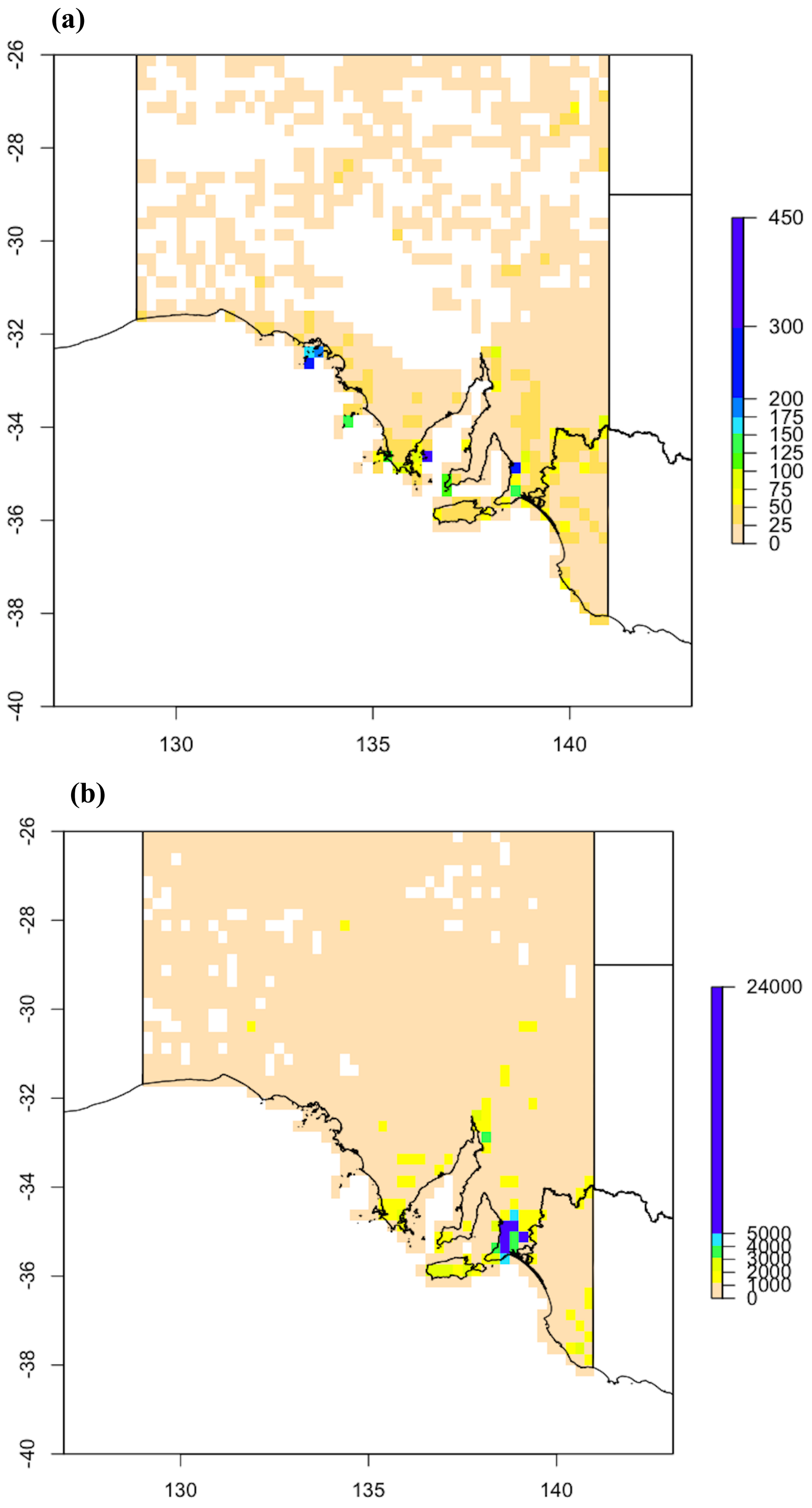

Figure B. Sampling intensity: (a) number of plots; **(b)** number of herbarium records.

(a)

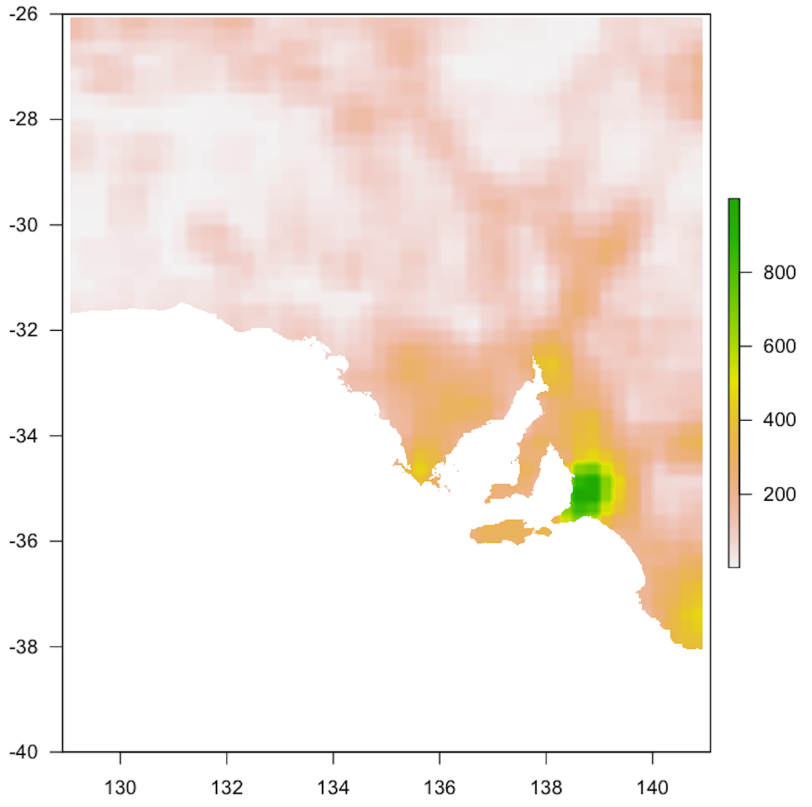

-26
-28
-30
-32
-34
-36
-38
-40
130
132
134
136
138
140
800
600
400
200

(b)

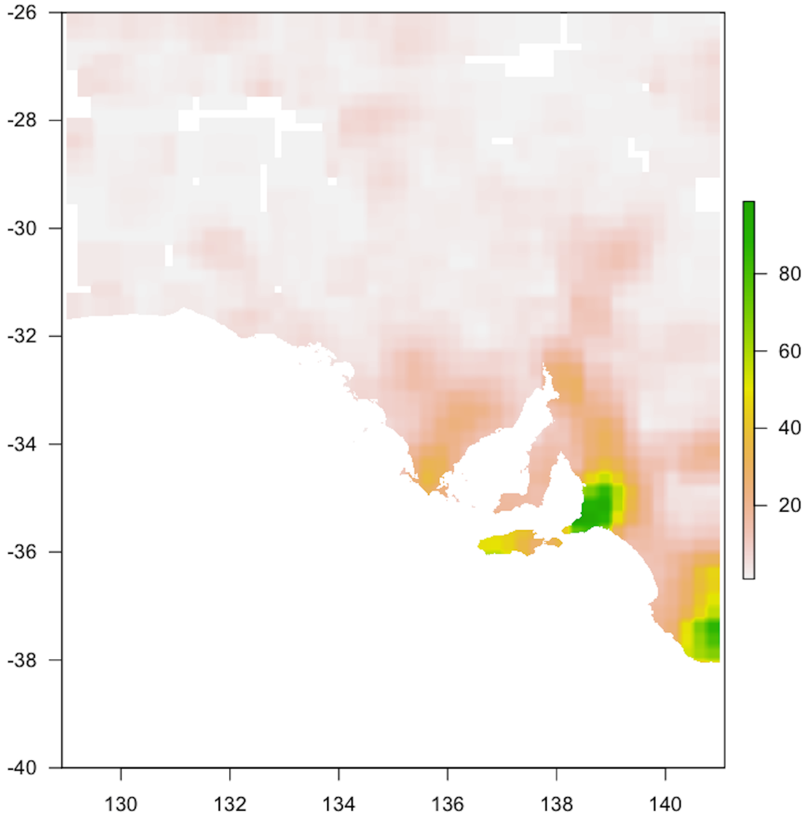

-26
-28
-30
-32
-34
-36
-38
-40
130
132
134
136
138
140
80
60
40
20

(c)

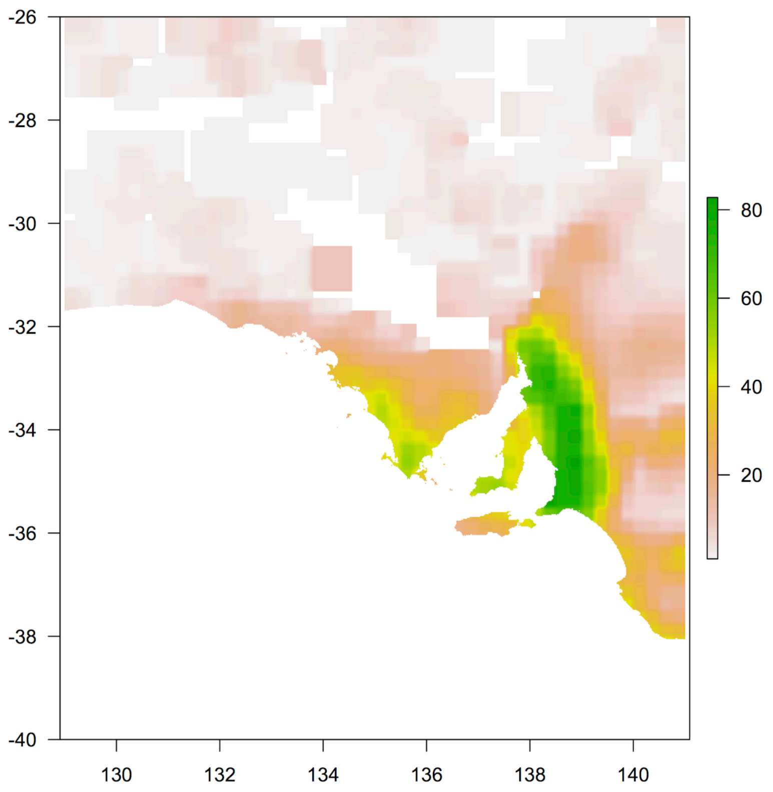

Figure C. ***Species richness*****: (a)** uncorrected *species richness* (herbarium records); **(b)** uncorrected *species richness* of conservation-dependent species (herbarium records)**; (c)** uncorrected *species richness* of introduced species (plots).

(a)

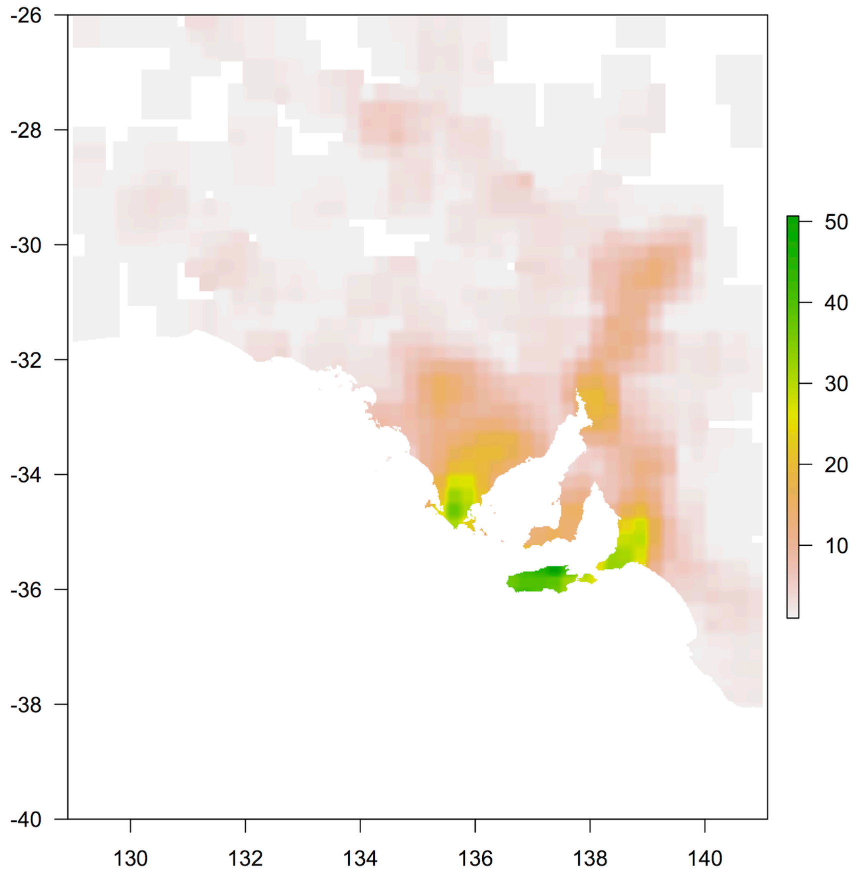
-26
-28
-30
-32
-34
-36
-38
-40
130
132
134
136
138
140
50
40
30
20
10

(b)

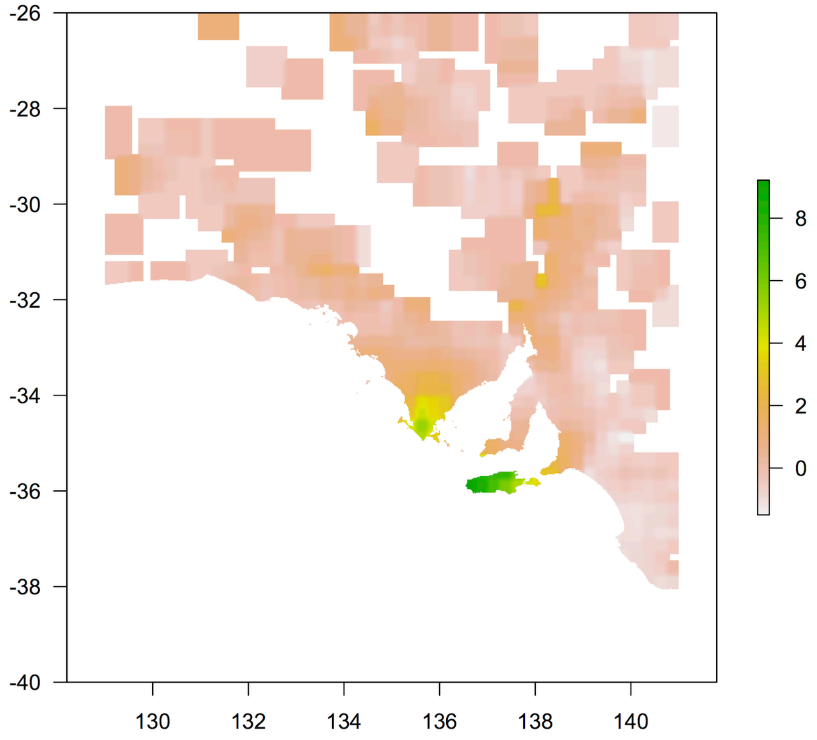
-26
-28
-30
-32
-34
-36
-38
-40
130
132
134
136
138
140
8
6
4
2
0

(c)

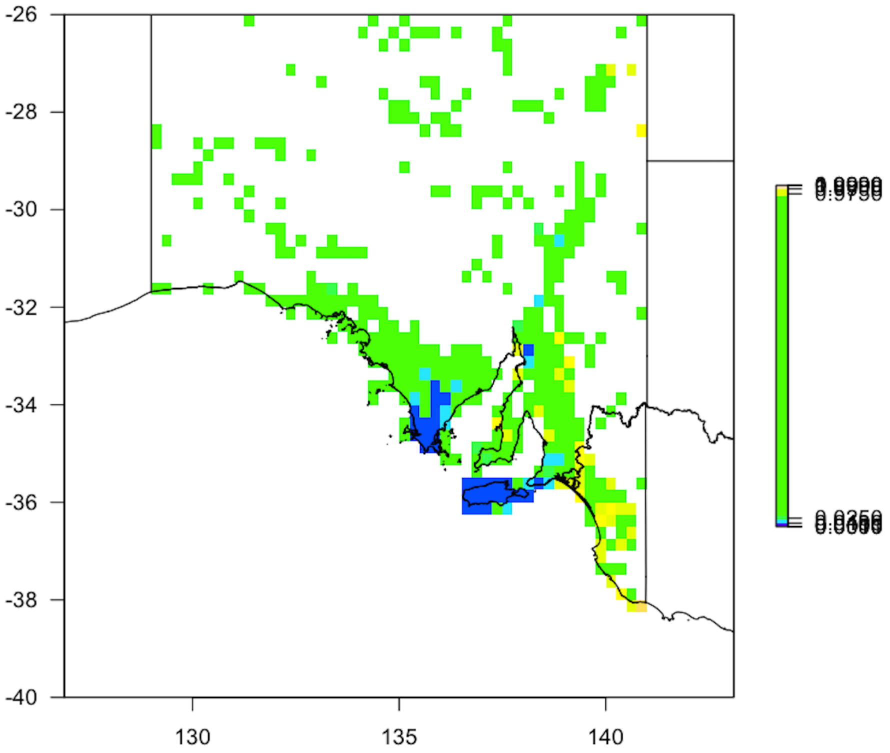

Figure D. ***Species richness*****: (a)** uncorrected *species richness* of categorical endemic species (herbarium records); **(b)** uncorrected *species richness* of categorical endemic species (plots); **(c)** 2-tailed statistical significance for higher or lower than expected richness of categorical endemic species.

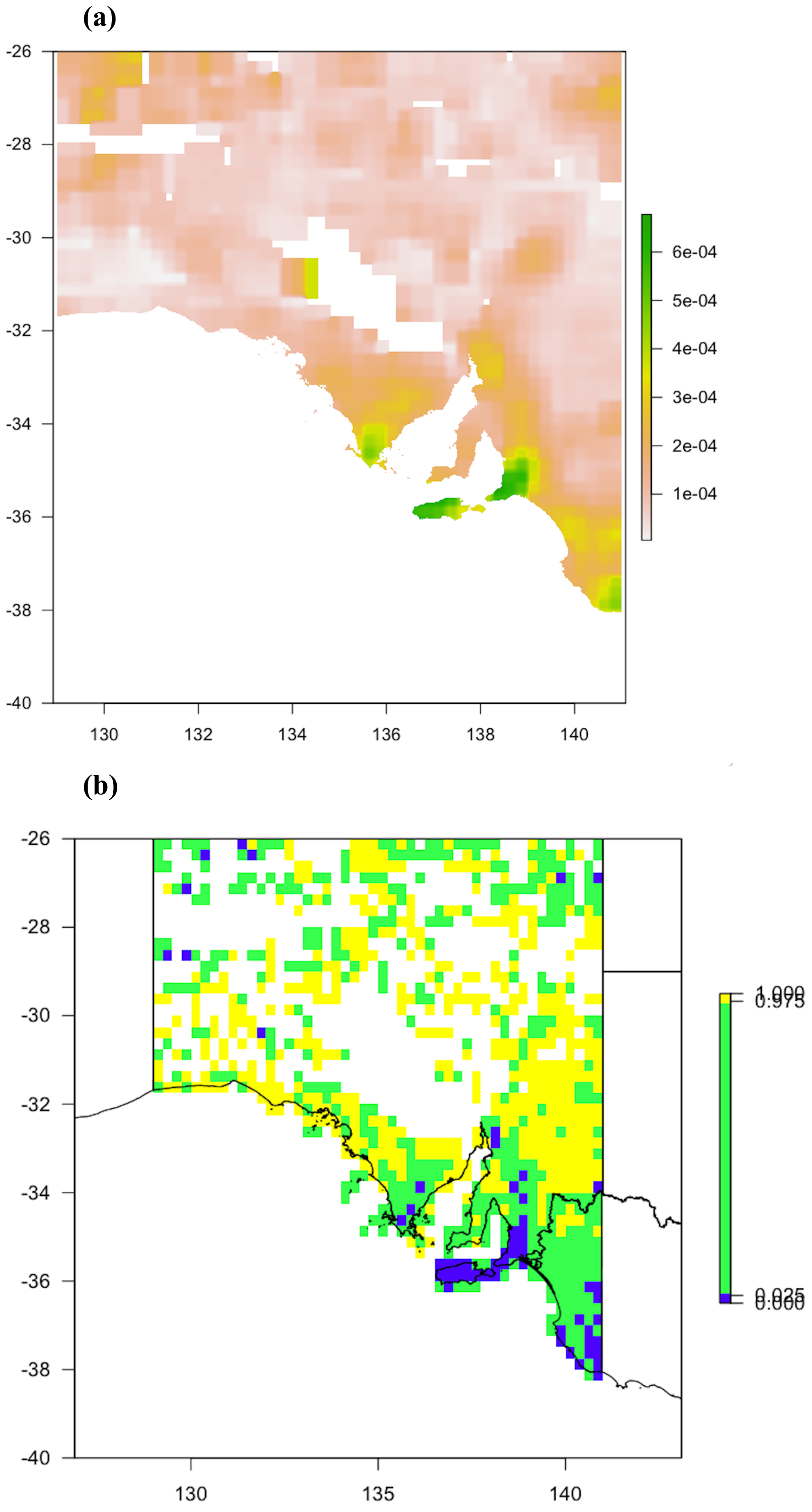

Figure E. ***Georeferenced weighted endemism***: **(a)** of native species (plots): raw values, not corrected for *species richness*/sampling; **(b)** 2-tailed statistical significance for higher or lower than expected endemism.

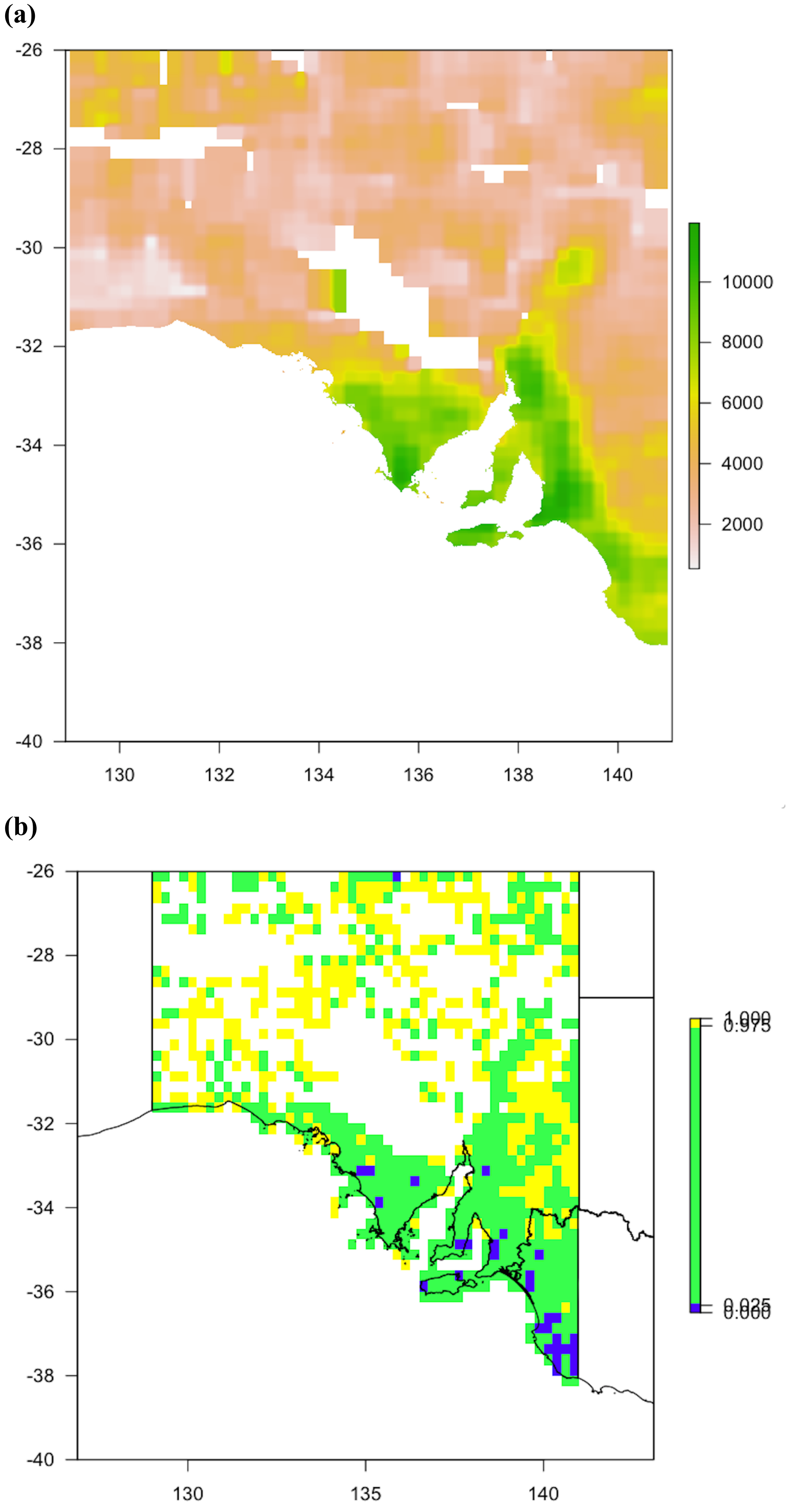

Figure F. ***Phylogenetic diversity***: **(a)** raw values, not corrected for *species richness*/sampling (plots); **(b)** 2-tailed statistical significance for higher or lower than expected.

(a)

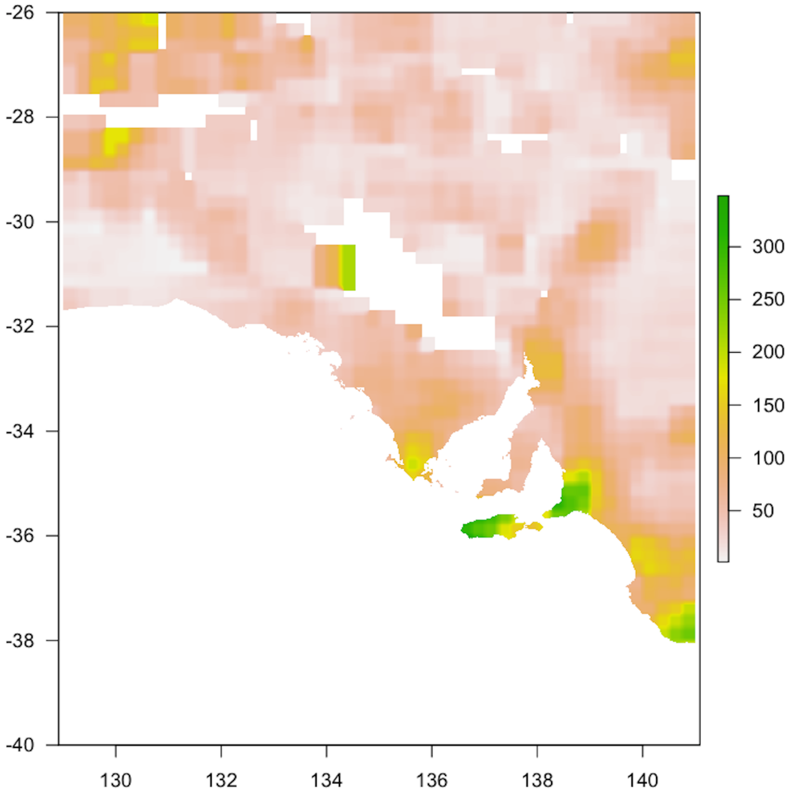

(b)

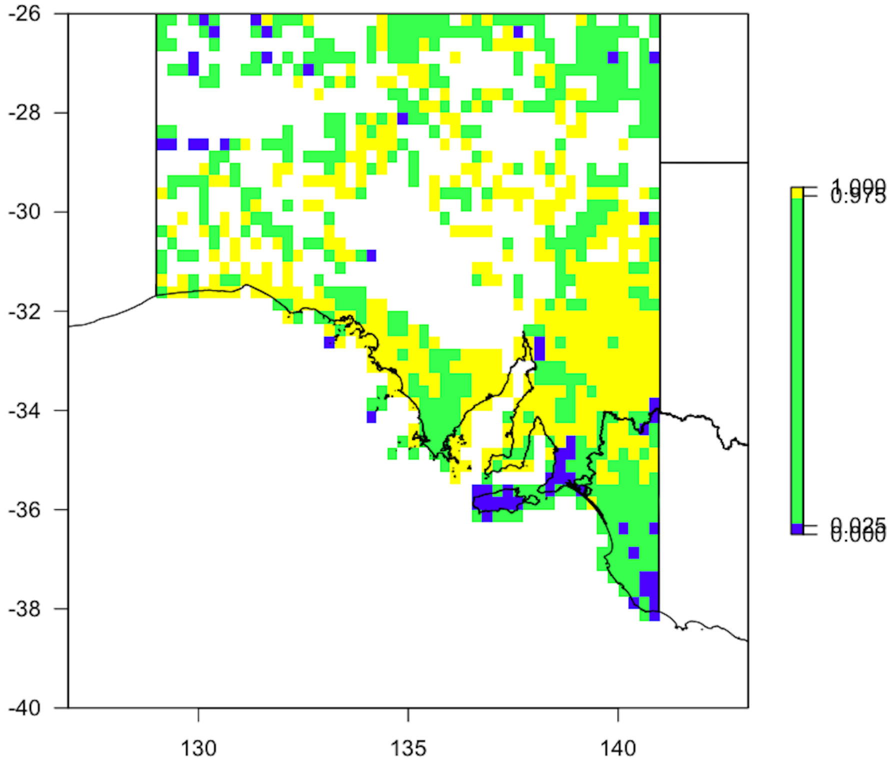

Figure G. ***Georeferenced phylogenetic endemism***: **(a)** raw values, not corrected for *species richness*/sampling (plots); **(b)** 2-tailed statistical significance for higher or lower than expected.

(a)

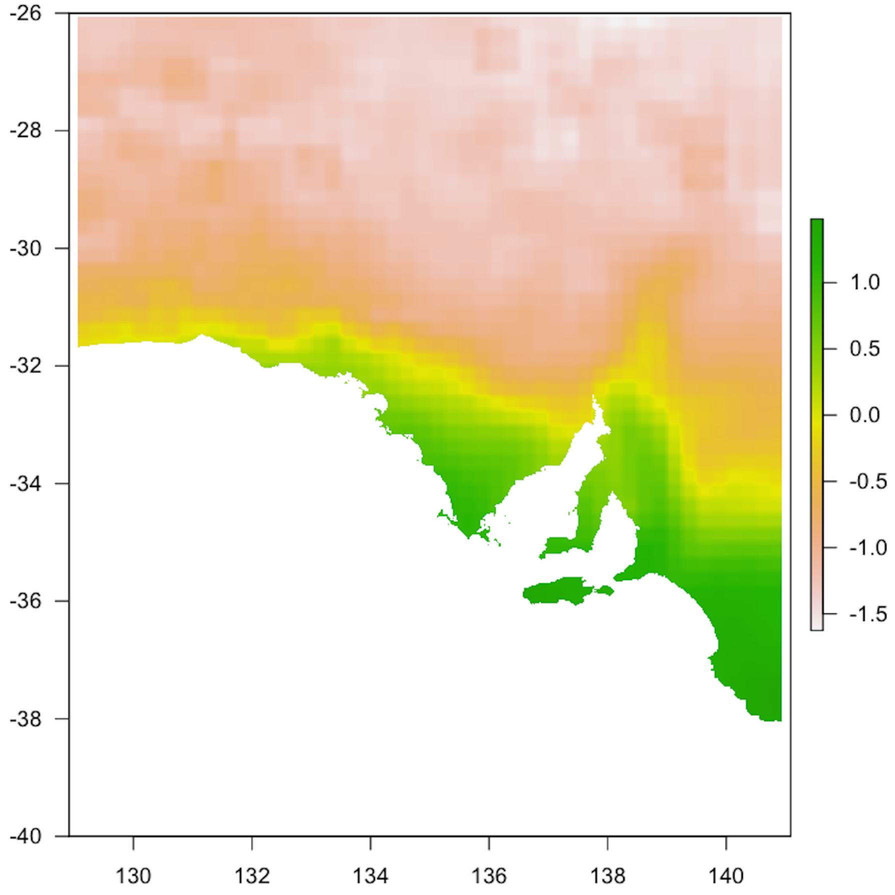
-26
-28
-30
-32
-34
-36
-38
-40
130
132
134
136
138
140
1.0
0.5
0.0
-0.5
-1.0
-1.5

(b)

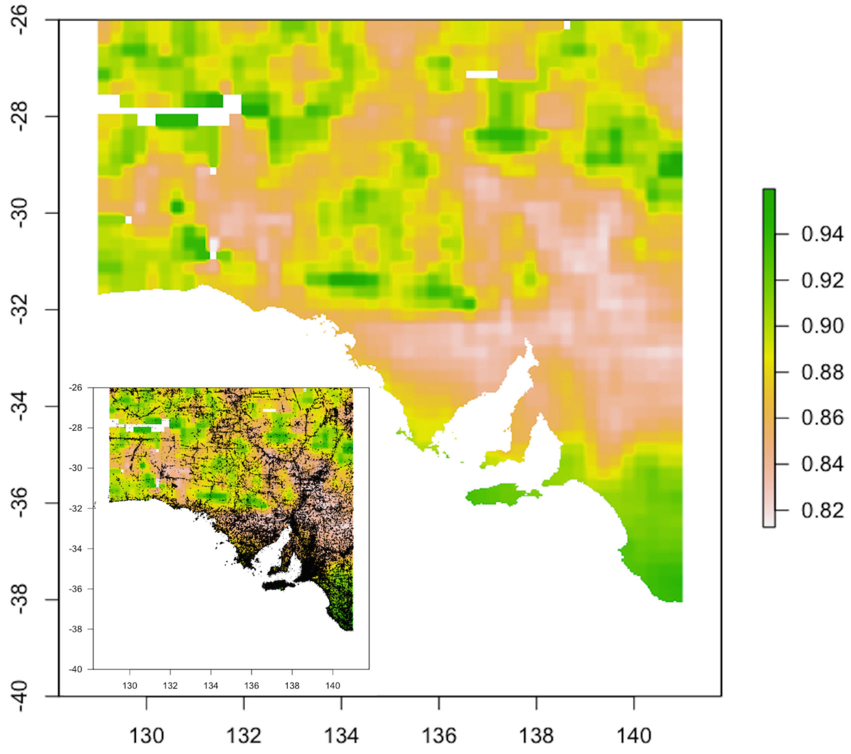
-26
-28
-30
-32
-34
-36
-38
-40
130
132
134
136
138
140
0.94
0.92
0.90
0.88
0.86
0.84
0.82

(c)

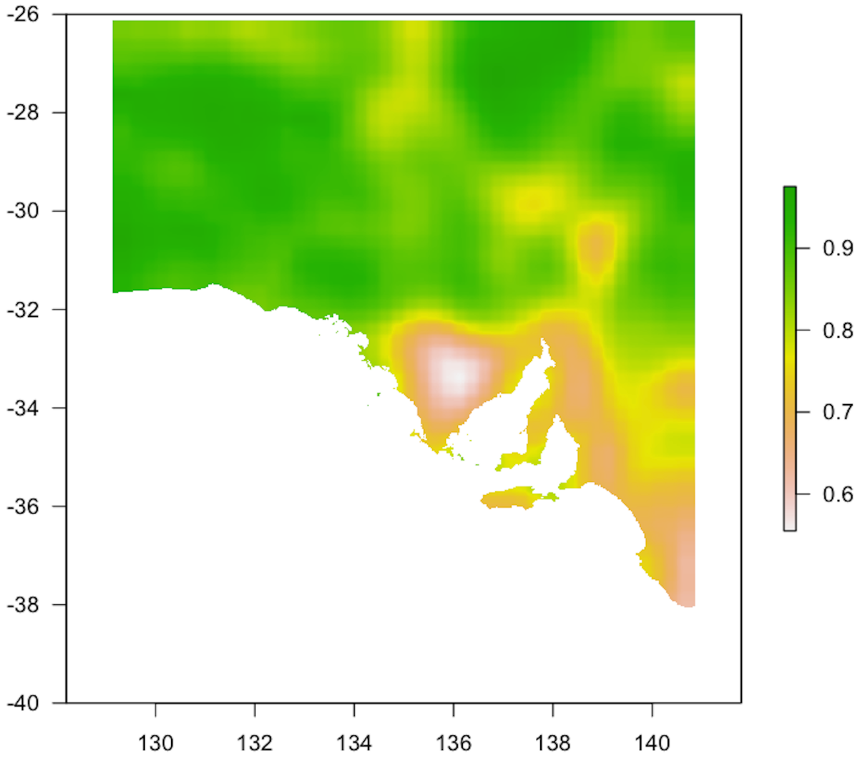

Figure H. Beta diversity analysis: (a) map with values representing position of grid cells along the first unconstrained Correspondence Analysis axis according to species composition; **(b)** measure of 'global' beta diversity with scores representing mean dissimilarity when compared to the rest of the state. Inset shows the same map with the data points mapped, showing that high global beta diversity in the outback is an artefact of under-sampled areas; **(c)** measure of local heterogeneity representing mean dissimilarity among grid cells in a moving window.

(a)

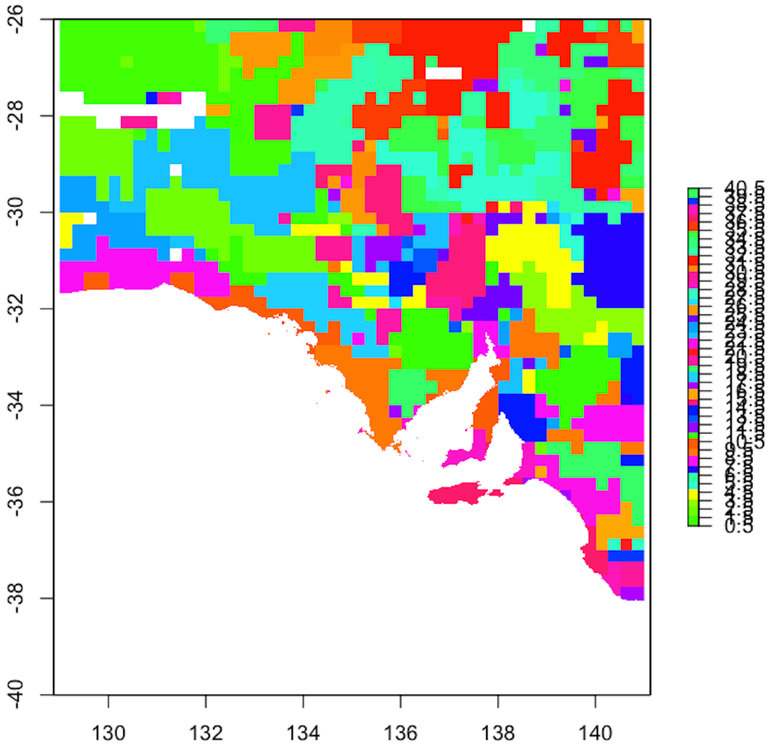
-26
-28
-30
-32
-34
-36
-38
-40
130
132
134
136
138
140
40.5
0.5

(b)

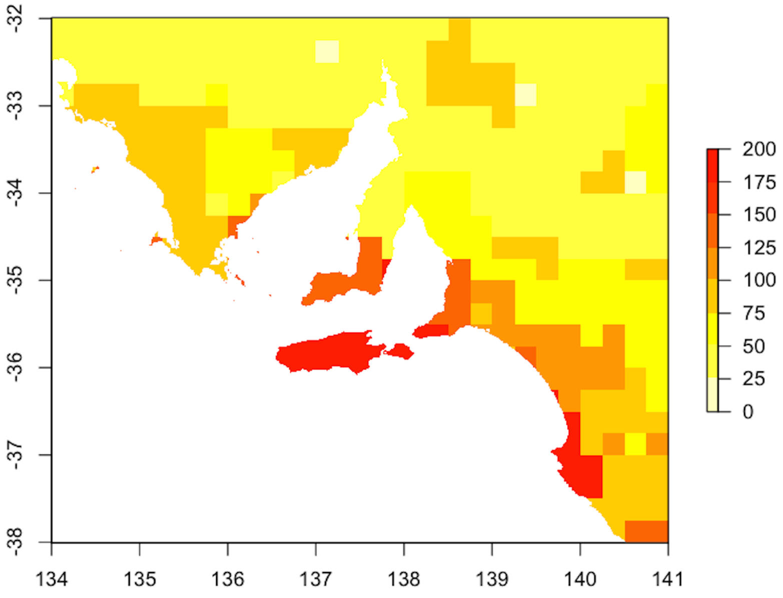
-32
-33
-34
-35
-36
-37
-38
134
135
136
137
138
139
140
141
200
175
150
125
100
75
50
25
0

(c)

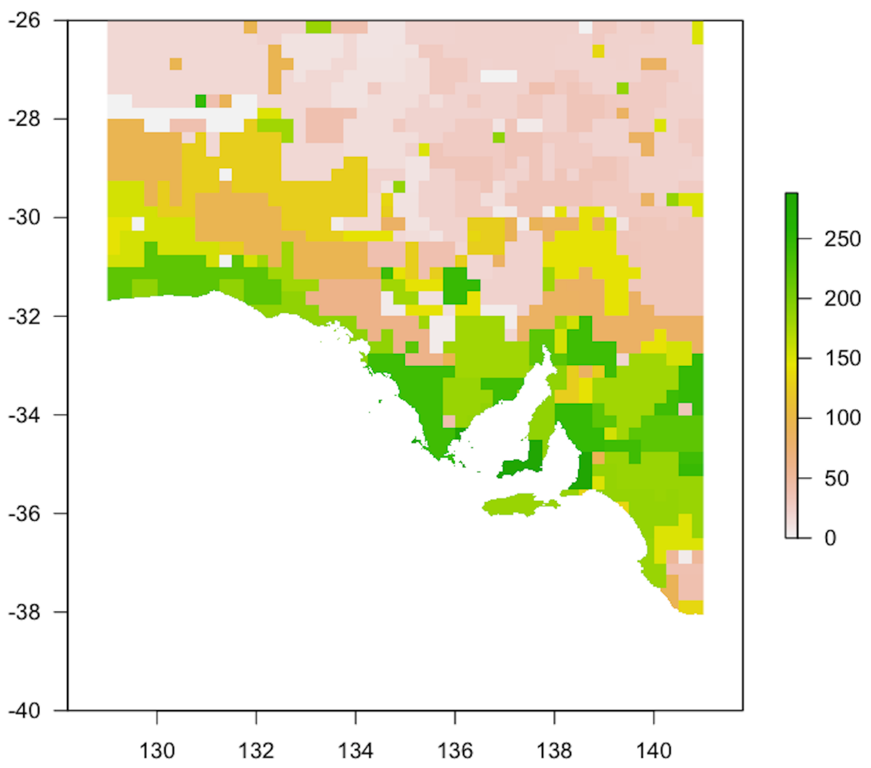

Figure I. Classification-based analysis: (a) map showing grid cells assigned to 40 clusters based on species composition; **(b)** zoomed in version of (a) with values for vegetation clusters given as the mean raw geographic phylogenetic endemism score for grid cells assigned to each cluster. The raw phylogenetic endemism metric combines elements of species richness, range restriction and phylogenetic diversity; **(c)** the classification map of (a) but with values showing the uncorrected richness of introduced plant species recorded within groups.

Table A. Most frequent species in 40 vegetation clusters shown in Figure I based on occurrences in grid cells assigned to clusters.

Species	Grid cell frequency	Vegetation cluster
Senna.artemisioides	0.89	1
Eremophila.latrobei	0.79	1
Dodonaea.viscosa	0.77	1
Aristida.contorta	0.77	1
Ptilotus.obovatus	0.75	1
Euphorbia.tannensis	0.75	1
Enchylaena.tomentosa	0.75	1
Senna.artemisioides	0.91	2
Enchylaena.tomentosa	0.84	2
Dodonaea.viscosa	0.78	2
Acacia.tetragonophylla	0.76	2
Santalum.acuminatum	0.73	2
Eucalyptus.socialis	0.73	2
Maireana.pyramidata	0.83	3
Calotis.hispidula	0.83	3
Atriplex.vesicaria	0.83	3
Sonchus.oleraceus	0.79	3
Sclerolaena.obliquicuspis	0.79	3
Enchylaena.tomentosa	0.79	3
Carrichtera.annua	0.79	3
Senna.artemisioides	0.91	4
Enchylaena.tomentosa	0.76	4
Austrostipa.nitida	0.76	4
Ptilotus.obovatus	0.7	4
Schismus.barbatus	0.67	4
Calotis.hispidula	0.67	4
Triraphis.mollis	0.79	5
Cullen.australasicum	0.79	5
Lotus.cruentus	0.74	5
Eragrostis.dielsii	0.74	5
Crotalaria.eremaea	0.74	5
Aristida.contorta	0.74	5
Senna.artemisioides	0.82	6
Eragrostis.dielsii	0.82	6
Sclerolaena.intricata	0.73	6
Salsola.australis	0.73	6
Eucalyptus.coolabah	0.73	6
Enchylaena.tomentosa	0.73	6
Aristida.contorta	0.73	6
Tetragonia.eremaea	0.94	7
Schismus.barbatus	0.92	7
Salsola.australis	0.92	7
Rhodanthe.moschata	0.89	7

Rhodanthe.floribunda	0.89	7
Atriplex.holocarpa	0.89	7
Sonchus.oleraceus	0.89	8
Sclerolaena.obliquicuspis	0.89	8
Austrostipa.nitida	0.89	8
Atriplex.vesicaria	0.89	8
Rytidosperma.caespitosum	0.86	8
Enchylaena.tomentosa	0.86	8
Carrichtera.annua	0.86	8
Melaleuca.lanceolata	0.84	9
Enchylaena.tomentosa	0.84	9
Rytidosperma.caespitosum	0.82	9
Daucus.glochidiatus	0.82	9
Sonchus.oleraceus	0.8	9
Oxalis.perennans	0.8	9
Bromus.rubens	0.8	9
Austrostipa.elegantissima	0.8	9
Melaleuca.lanceolata	0.96	10
Geijera.linearifolia	0.94	10
Exocarpos.aphyllus	0.9	10
Enchylaena.tomentosa	0.9	10
Eucalyptus.oleosa	0.86	10
Enchylaena.tomentosa	0.91	11
Senna.artemisioides	0.9	11
Atriplex.vesicaria	0.86	11
Sclerolaena.obliquicuspis	0.84	11
Maireana.sedifolia	0.81	11
Carrichtera.annua	0.81	11
Convolvulus.remotus	0.67	12
Sonchus.oleraceus	0.58	12
Senna.artemisioides	0.58	12
Rytidosperma.caespitosum	0.58	12
Austrostipa.scabra	0.58	12
Austrostipa.nitida	0.58	12
Senna.artemisioides	0.87	13
Pycnosorus.pleiocephalus	0.73	13
Hypochaeris.glabra	0.73	13
Exocarpos.aphyllus	0.73	13
Daucus.glochidiatus	0.73	13
Actinobole.uliginosum	0.73	13
Eucalyptus.socialis	0.83	14
Chenopodium.desertorum	0.83	14
Enchylaena.tomentosa	0.79	14
Hypochaeris.glabra	0.76	14
Senna.artemisioides	0.72	14
Einadia.nutans	0.72	14
Atriplex.vesicaria	0.91	15

Senna.artemisioides	0.87	15
Maireana.aphylla	0.83	15
Sclerolaena.ventricosa	0.74	15
Rhagodia.spinescens	0.74	15
Eragrostis.setifolia	0.74	15
Enchylaena.tomentosa	0.74	15
Atriplex.lindleyi	0.74	15
Sonchus.oleraceus	0.63	16
Enchylaena.tomentosa	0.53	16
Daucus.glochidiatus	0.53	16
Lachnagrostis.filiformis	0.47	16
Teucrium.racemosum	0.42	16
Pimelea.glauca	0.42	16
Lepidosperma.concavum	0.42	16
Lepidosperma.carphoides	0.42	16
Juncus.kraussii	0.42	16
Eucalyptus.fasciculosa	0.42	16
Dillwynia.hispida	0.42	16
Crassula.decumbens	0.42	16
Calytrix.tetragona	0.42	16
Billardiera.cymosa	0.42	16
Asparagus.asparagoides	0.42	16
Allocasuarina.muelleriana	0.42	16
Acacia.pycnantha	0.42	16
Sonchus.oleraceus	0.53	17
Oxalis.perennans	0.53	17
Daucus.glochidiatus	0.53	17
Bursaria.spinosa	0.53	17
Pimelea.glauca	0.47	17
Melaleuca.lanceolata	0.47	17
Hypochaeris.glabra	0.47	17
Dianella.brevicaulis	0.47	17
Clematis.microphylla	0.47	17
Carduus.tenuiflorus	0.47	17
Astroloma.humifusum	0.47	17
Olearia.muelleri	0.95	18
Westringia.rigida	0.91	18
Rhagodia.crassifolia	0.91	18
Dodonaea.bursariifolia	0.91	18
Geijera.linearifolia	0.86	18
Exocarpos.aphyllus	0.86	18
Eucalyptus.oleosa	0.86	18
Eucalyptus.gracilis	0.86	18
Eremophila.weldii	0.86	18
Eremophila.glabra	0.86	18
Dodonaea.stenozyga	0.86	18
Dianella.revoluta	0.86	18

Melaleuca.lanceolata	0.91	19
Eucalyptus.leptophylla	0.91	19
Eucalyptus.incrassata	0.91	19
Billardiera.cymosa	0.91	19
Lepidosperma.viscidum	0.88	19
Ptilotus.obovatus	0.8	20
Calotis.hispidula	0.8	20
Zygophyllum.aurantiacum	0.73	20
Senna.artemisioides	0.73	20
Podolepis.capillaris	0.73	20
Eucalyptus.socialis	0.73	20
Dodonaea.viscosa	0.73	20
Senna.artemisioides	0.6	21
Atriplex.lindleyi	0.6	21
Sonchus.oleraceus	0.53	21
Lepidium.phlebopetalum	0.53	21
Triraphis.mollis	0.47	21
Stemodia.florulenta	0.47	21
Solanum.ellipticum	0.47	21
Sclerolaena.diacantha	0.47	21
Rhodanthe.moschata	0.47	21
Plantago.drummondii	0.47	21
Leiocarpa.leptolepis	0.47	21
Daucus.glochidiatus	0.47	21
Cullen.australasicum	0.47	21
Calocephalus.platycephalus	0.47	21
Atriplex.holocarpa	0.47	21
Thysanotus.patersonii	0.64	22
Sonchus.oleraceus	0.64	22
Melaleuca.lanceolata	0.64	22
Hypochaeris.glabra	0.64	22
Helichrysum.leucopsideum	0.64	22
Exocarpos.sparteus	0.64	22
Eucalyptus.leptophylla	0.64	22
Eucalyptus.incrassata	0.64	22
Acacia.spinescens	0.64	22
Senna.artemisioides	0.84	23
Dodonaea.viscosa	0.73	23
Enchylaena.tomentosa	0.71	23
Acacia.aneura	0.7	23
Sclerolaena.diacantha	0.68	23
Enchylaena.tomentosa	0.83	24
Senna.artemisioides	0.81	24
Pittosporum.angustifolium	0.71	24
Rhagodia.spinescens	0.69	24
Alectryon.oleifolius	0.69	24
Enchylaena.tomentosa	0.74	25

Senna.artemisioides	0.67	25
Lotus.cruentus	0.63	25
Eremophila.longifolia	0.63	25
Sida.fibulifera	0.59	25
Pterocaulon.sphacelatum	0.59	25
Nicotiana.velutina	0.59	25
Calotis.hispidula	0.59	25
Atriplex.vesicaria	0.59	25
Senna.artemisioides	0.87	26
Eremophila.latrobei	0.78	26
Aristida.contorta	0.78	26
Monachather.paradoxus	0.7	26
Acacia.aneura	0.7	26
Sonchus.oleraceus	0.79	27
Solanum.ellipticum	0.64	27
Oxalis.perennans	0.57	27
Dodonaea.viscosa	0.57	27
Convolvulus.remotus	0.57	27
Daucus.glochidiatus	0.78	28
Dodonaea.viscosa	0.67	28
Xanthorrhoea.caespitosa	0.56	28
Thysanotus.patersonii	0.56	28
Thomasia.petalocalyx	0.56	28
Senecio.picridioides	0.56	28
Rytidosperma.geniculatum	0.56	28
Pyrorchis.nigricans	0.56	28
Oxalis.perennans	0.56	28
Melaleuca.lanceolata	0.56	28
Melaleuca.brevifolia	0.56	28
Leptospermum.myrsinoides	0.56	28
Lepidosperma.viscidum	0.56	28
Lepidosperma.congestum	0.56	28
Lepidosperma.carphoides	0.56	28
Juncus.kraussii	0.56	28
Isolepis.nodosa	0.56	28
Isolepis.marginata	0.56	28
Hypolaena.fastigiata	0.56	28
Hypochaeris.radicata	0.56	28
Hypochaeris.glabra	0.56	28
Hibbertia.sericea	0.56	28
Goodenia.geniculata	0.56	28
Gonocarpus.tetragynus	0.56	28
Eucalyptus.siderophloia	0.56	28
Eucalyptus.leucoxylon	0.56	28
Eucalyptus.fasciculosa	0.56	28
Erodium.cicutarium	0.56	28
Drosera.whittakeri	0.56	28

Dillwynia.sericea	0.56	28
Dianella.caerulea	0.56	28
Cynoglossum.australe	0.56	28
Crassula.decumbens	0.56	28
Crassula.colorata	0.56	28
Comesperma.volubile	0.56	28
Comesperma.calymega	0.56	28
Cirsium.vulgare	0.56	28
Chamaescilla.corymbosa	0.56	28
Centrolepis.polygyna	0.56	28
Cassytha.glabella	0.56	28
Calytrix.tetragona	0.56	28
Caladenia.carnea	0.56	28
Burchardia.umbellata	0.56	28
Briza.minor	0.56	28
Billardiera.cymosa	0.56	28
Baumea.juncea	0.56	28
Banksia.ornata	0.56	28
Banksia.marginata	0.56	28
Astroloma.humifusum	0.56	28
Arthropodium.strictum	0.56	28
Amyema.miquelii	0.56	28
Acacia.pycnantha	0.56	28
Acacia.myrtifolia	0.56	28
Tetragonia.implexicoma	1	29
Threlkeldia.diffusa	0.97	29
Rhagodia.candolleana	0.94	29
Olearia.axillaris	0.94	29
Senecio.pinnatifolius	0.91	29
Disphyma.crassifolium	0.91	29
Carpobrotus.rossii	0.91	29
Atriplex.cinerea	0.91	29
Senna.artemisioides	0.74	30
Ptilotus.polystachyus	0.56	30
Ptilotus.obovatus	0.56	30
Eremophila.latrobei	0.56	30
Enchylaena.tomentosa	0.56	30
Trianthema.triquetra	0.87	31
Eremophila.latrobei	0.87	31
Senna.artemisioides	0.78	31
Goodenia.lunata	0.78	31
Eucalyptus.coolabah	0.78	31
Eragrostis.setifolia	0.78	31
Atriplex.vesicaria	0.78	31
Polycalymma.stuartii	0.94	32
Salsola.australis	0.85	32
Crotalaria.eremaea	0.83	32

Zygochloa.paradoxa	0.79	32
Enchylaena.tomentosa	0.77	32
Senna.artemisioides	0.88	33
Atriplex.vesicaria	0.85	33
Salsola.australis	0.77	33
Acacia.tetragonophylla	0.73	33
Eragrostis.setifolia	0.69	33
Enneapogon.polyphyllus	0.69	33
Dissocarpus.paradoxus	0.69	33
Senna.artemisioides	0.86	34
Eragrostis.dielsii	0.77	34
Crotalaria.eremaea	0.77	34
Aristida.contorta	0.7	34
Enchylaena.tomentosa	0.67	34
Salsola.australis	0.81	35
Eragrostis.setifolia	0.81	35
Sclerolaena.intricata	0.71	35
Eragrostis.dielsii	0.71	35
Enchylaena.tomentosa	0.71	35
Atriplex.spongiosa	0.71	35
Leiocarpa.leptolepis	0.77	36
Senna.artemisioides	0.73	36
Enchylaena.tomentosa	0.73	36
Eucalyptus.coolabah	0.7	36
Eragrostis.setifolia	0.7	36
Dianella.brevicaulis	0.87	37
Daucus.glochidiatus	0.87	37
Sonchus.oleraceus	0.83	37
Rhagodia.candolleana	0.83	37
Poa.poiformis	0.83	37
Olearia.axillaris	0.83	37
Leucopogon.parviflorus	0.83	37
Austrostipa.flavescens	0.83	37
Sonchus.oleraceus	0.57	38
Daucus.glochidiatus	0.57	38
Thysanotus.patersonii	0.5	38
Senna.artemisioides	0.5	38
Rhagodia.candolleana	0.5	38
Pomaderris.paniculosa	0.5	38
Pimelea.glauca	0.5	38
Oxalis.perennans	0.5	38
Melaleuca.lanceolata	0.5	38
Lomandra.micrantha	0.5	38
Clematis.microphylla	0.5	38
Bursaria.spinosa	0.5	38
Acrotriche.cordata	0.5	38
Chrysocephalum.apiculatum	0.53	39

Sonchus.oleraceus	0.47	39
Senna.artemisioides	0.47	39
Helichrysum.luteoalbum	0.42	39
Daucus.glochidiatus	0.42	39
Aristida.contorta	0.42	39
Polycalymma.stuartii	0.73	40
Crotalaria.eremaea	0.73	40
Zygophyllum.howittii	0.67	40
Senecio.gregorii	0.67	40
Plantago.drummondii	0.67	40
Atriplex.velutinella	0.67	40
